# Metabolic reprogramming of interleukin-17-producing γδ T cells promotes ACC1-mediated de novo lipogenesis under psoriatic conditions

**DOI:** 10.1038/s42255-025-01276-z

**Published:** 2025-05-13

**Authors:** Yu-San Kao, Mario Lauterbach, Aleksandra Lopez Krol, Ute Distler, Gloria Janet Godoy, Matthias Klein, Rafael Jose Argüello, Fatima Boukhallouk, Sara Vallejo Fuente, Kathrin Luise Braband, Assel Nurbekova, Monica Romero, Panagiota Mamareli, Luana Silva, Luis Eduardo Alves Damasceno, Francesca Rampoldi, Luciana Berod, Lydia Lynch, Karsten Hiller, Tim Sparwasser

**Affiliations:** 1 Institute of Medical Microbiology and Hygiene, University Medical Center of the Johannes Gutenberg University Mainz, Mainz, Germany; 2 Department of Bioinformatics and Biochemistry, Braunschweig Integrated Centre of Systems Biology, Technical University of Braunschweig, Braunschweig, Germany; 3 Research Center for Immunotherapy (FZI), University Medical Center of the Johannes Gutenberg University Mainz, Mainz, Germany; 4 Institute for Immunology, University Medical Center of the Johannes Gutenberg-University Mainz, Mainz, Germany; 5 Aix Marseille University, CNRS, INSERM, CIML, Centre d’Immunologie de Marseille–Luminy, Marseille, France; 6 Institute of Molecular Medicine, University Medical Center of the Johannes Gutenberg University Mainz, Mainz, Germany; 7 Molecular Biology, Princeton University, Princeton, NJ USA; 8 Present Address: Ludwig Cancer Research Institute, Princeton Branch, Princeton University, Princeton, NJ USA; 9 Present Address: Institute of Molecular Medicine, University Medical Center of the Johannes Gutenberg University Mainz, Mainz, Germany; 10 Present Address: Institute for Immunology, University Medical Center of the Johannes Gutenberg University Mainz, Mainz, Germany; 11 Present Address: Center for Research in Inflammatory Diseases (CRID), Department of Pharmacology, Ribeirao Preto Medical School, University of Sao Paulo, São Paulo, Brazil; 12 Present Address: DKFZ German Cancer Research Center, Heidelberg, Germany

**Keywords:** Autoimmunity, Gammadelta T cells, Metabolism, Metabolomics, Fatty acids

## Abstract

Metabolic reprogramming determines γδ T cell fate during thymic development; however, the metabolic requirements of interleukin (IL)-17A-producing γδ T cells (γδT17 cells) under psoriatic conditions are unclear. Combining high-throughput techniques, including RNA sequencing, SCENITH, proteomics and stable isotope tracing, we demonstrated that psoriatic inflammation caused γδT17 cells to switch toward aerobic glycolysis. Under psoriatic conditions, γδT17 cells upregulated ATP-citrate synthase to convert citrate to acetyl-CoA, linking carbohydrate metabolism and fatty acid synthesis (FAS). Accordingly, we used a pharmacological inhibitor, Soraphen A, which blocks acetyl-CoA carboxylase (ACC), to impair FAS in γδT17 cells, reducing their intracellular lipid stores and ability to produce IL-17A under psoriatic conditions in vitro. We pinpointed the pathogenic role of ACC1 in γδT17 cells in vivo by genetic ablation, ameliorating inflammation in a psoriatic mouse model. Furthermore, ACC inhibition limited human IL-17A-producing γδT17 cells. Targeting ACC1 to attenuate pathogenic γδT17 cell function has important implications for psoriasis management.

## Abstract (web summary)

Kao et al. provide insight into the metabolic requirements of IL-17A-producing γδ T cells, offering an actionable target to treat psoriasis by inhibiting fatty acid synthesis.

## Main

Psoriasis is an immune-mediated inflammatory disease driven by abnormal IL-17A–IL-23 axis activation^1–4^. IL-17A, produced by activated T cells, triggers keratinocyte hyperproliferation and massive type 17 immune cell recruitment^1–3^, resulting in psoriatic skin inflammation. IL-17^+^CD4^+^ T helper (T_H_)17 cells are the primary IL-17A producers in response to self-antigens^5^. Studies have linked dysregulated lipid metabolism to pathogenic T_H_17 cells in multiple inflammatory diseases, including psoriasis^6–9^. Accordingly, we have shown that targeting the rate-limiting enzyme of FAS, acetyl-CoA carboxylase 1 (ACC1), in αβ T cells attenuated psoriatic skin inflammation in a mouse model of imiquimod (IMQ)-induced psoriasis^9^. Nevertheless, IL-17A-producing γδT17 cells were reported to be significantly increased in the psoriatic skin lesions of patients^10–18^. Dermal γδ T cells constitutively express the IL-23 receptor (IL-23R) and represent a major IL-17A source in response to IL-23 directly^10–18^. Thus, understanding the metabolic mechanisms determining IL-17A-producing γδT17 cell effector function is of great interest.

γδ T cells are a unique T cell subpopulation enriched in multiple peripheral tissues, providing localized immune responsiveness at anatomical sites that are inadequately served by αβ T cells^18^. Unlike αβ T cells, γδ T cells can produce large quantities of cytokines (for example, IL-17A) independently of major histocompatibility complex restriction; thus, they have essential roles in inflammatory diseases such as psoriasis^10,13,14,18^. Successful thymic selection generates γδ T cells with unique γδ T cell receptors (TCRs), which are less diverse than αβ TCRs^19,20^. At this point, naive γδ T cells can either leave the thymus to the circulatory system and secondary lymphoid organs or undergo further differentiation and commit to the interferon-γ (IFNγ)-producing or IL-17-producing lineage, γδIFN, and γδT17 cells^20^. Most murine γδ T cells are pre-programmed to develop into the γδT17 or γδIFN functional subsets^20^, following a step-wise metabolic programme during thymic development^21^. Early CD24^+^ γδT precursors upregulate their mitochondrial membrane potential (MMP) to develop into γδT17 cells^21^. Meanwhile, γδT17 cells maintain high mitochondrial dependence acquired in the thymus even after reaching peripheral lymphoid organs^21^.

Psoriatic inflammation selectively promotes the migration and expansion of Vγ4^+^γδ T cells in mice^15–18,22^. Accordingly, inhibiting mitochondrial translation effectively reduces Vγ4^+^γδT17 cells, attenuating psoriatic inflammation^23^. IL-23 or IL-1β alone barely elicits dermal γδ T cells to produce IL-17A^24–26^. However, combining IL-23 and IL-1β stimulates dermal γδ T cells to produce large amounts of IL-17A^25,26^. IL-23R and IL-1R-deficient dermal γδ T cells fail to produce IL-17A^25^. Gardiquimod (a TLR7 agonist) induced IL-17A production in murine skin cell suspension containing dermal γδ T cells, and that effect was completely abolished in the γδ T cells from IL-1R-knockout (KO) mice^10^. Furthermore, the psoriatic inflammation mediated by γδT17 cells is mainly dependent on their response to cytokines, as demonstrated by the attenuated inflammation induced by IMQ in mice bearing IL-1R-deficient γδ T cells compared to wild-type (WT) littermates^26^. Nevertheless, it remains unclear whether psoriatic inflammation (that is, increased IL-1β and IL-23 levels^24–27^) induces γδT17 cells to engage other metabolic pathways. In addition to the role of Vγ4^+^γδ T cells in mice, human γδ T cells have been shown to express IL-17A in situ in psoriatic skin lesions from patients^10^. However, the link between the usage of the γδ TCR and IL-17A-producing phenotype in tissue remains to be further investigated in humans^18^.

γδT17 cells have high lipid storage and can further increase their intracellular lipid content under psoriatic conditions^21^. However, whether γδT17 cells can orchestrate de novo FAS to meet their high lipid demand under psoriatic conditions is unknown. Typically, pyruvate produced by the glycolytic pathway is converted into acetyl-CoA in the mitochondria before entering the tricarboxylic acid (TCA) cycle as citrate^28–30^. During T_H_ cell differentiation, citrate is transported to the cytosol by the citrate carrier^6,31,32^. ATP-citrate lyase (ACLY) converts citrate to acetyl-CoA and oxaloacetate^30,33^. Acetyl-CoA can then be converted to malonyl-CoA, used for de novo FAS mediated by the rate-limiting enzyme ACC1^34,35^. Subsequently, fatty acid synthase (FASN) uses seven malonyl-CoA molecules and one acetyl-CoA primer to synthesize palmitate^30^. Although two ACC isoforms, ACC1 and ACC2, catalyse the ATP-dependent carboxylation of acetyl-CoA to malonyl-CoA^36,37^, only ACC1 is crucial for de novo FAS in the cytosol. Previous work showed that pharmacological ACC inhibition prevented the development of T_H_17-mediated experimental autoimmune encephalitis and limited the severity of intestinal infections^6,7,38^. These findings imply that inhibiting ACC1-mediated FAS represents a feasible therapeutic strategy for psoriasis.

We previously demonstrated that inhibiting ACC1 in αβ T cells attenuated psoriatic skin inflammation in the IMQ model by limiting T_H_17 and Tc17 (an IL-17-producing subset of CD8^+^ T cells) numbers without affecting γδT17 cells^9^. Residual γδT17 cells significantly contributed to IL-17-mediated psoriatic inflammation in the IMQ model^9,13,15,16^. Nevertheless, how the metabolic dysregulation of γδT17 cells contributes to psoriasis pathogenesis remains poorly understood. Therefore, the present study aimed to investigate the metabolic requirements for γδT17 cells and determine the role of ACC1-mediated FAS in psoriatic inflammation.

## Results

### Metabolic reprogramming in γδT17 cells upon psoriatic condition

**Fig. 1:**
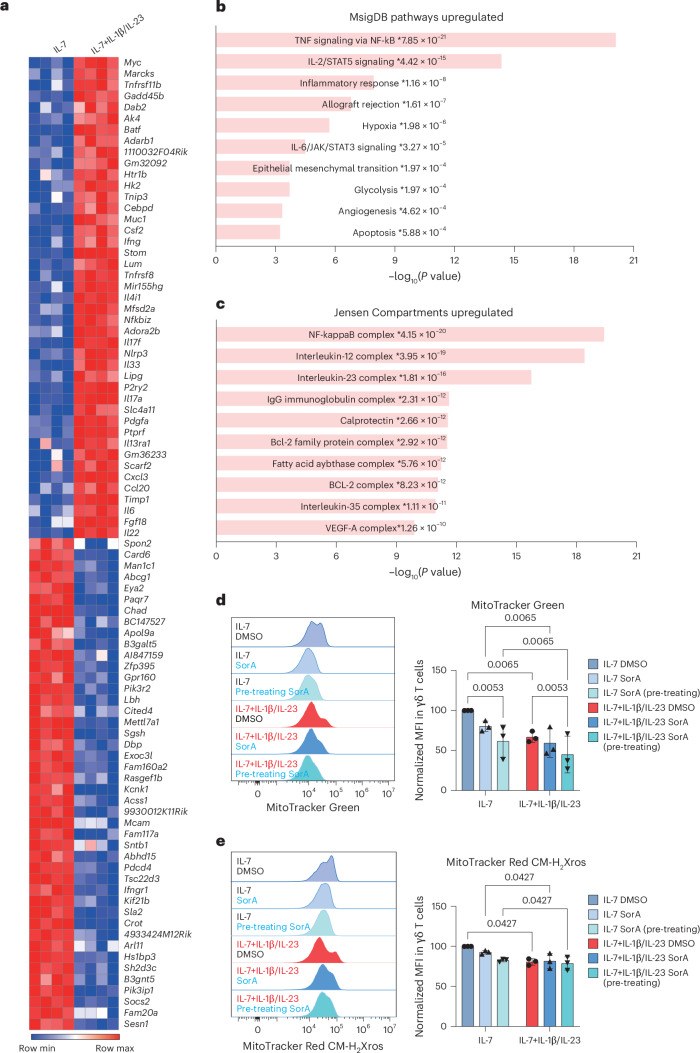
γδT17 cells undergo metabolic reprogramming toward aerobic glycolysis under psoriatic conditions. In vitro-expanded γδT17 cells (lineage^−^γδTCR^+^CD27^−^) were re-seeded on day 9 and stimulated with IL-7 in the presence (psoriatic conditions) or absence (homeostatic conditions) of IL-1β and IL-23 for 3 h. **a**, RNA-seq heatmap showing the upregulated (red) and downregulated (blue) genes (fold change > 12) in γδT17 cells under psoriatic versus homeostatic conditions. **b**, MsigDB pathway analysis of genes upregulated in γδT17 cells under psoriatic conditions. **c**, Jensen Compartment analysis of genes upregulated in γδT17 cells under psoriatic conditions. *P* values are obtained by the Benjamini–Hochberg-corrected *t*-test for **b** and **c**. **d**,**e**, In vitro*-*expanded γδT17 cells were sorted on day 6 and cultured with IL-7 for 3 days in the presence or absence of 1,000 nM SorA. On day 9, cells were re-seeded and stimulated with IL-7 either alone or combined with IL-1β and IL-23 for 24 h and treated with DMSO or 1,000 nM SorA. Representative flow cytometry histograms (left) and a summary graph (right) showing MitoTracker Green staining (**d**) and MitoTracker Red CM-H_2_Xros staining (**e**) in γδT17 cells under indicated conditions. Pooled means of normalized mean fluorescence intensities (MFIs) from three independent experiments are shown. Error bars, s.d. *P* values were obtained using two-way ANOVA for **d** and **e**.

**Extended Data Fig. 1:**
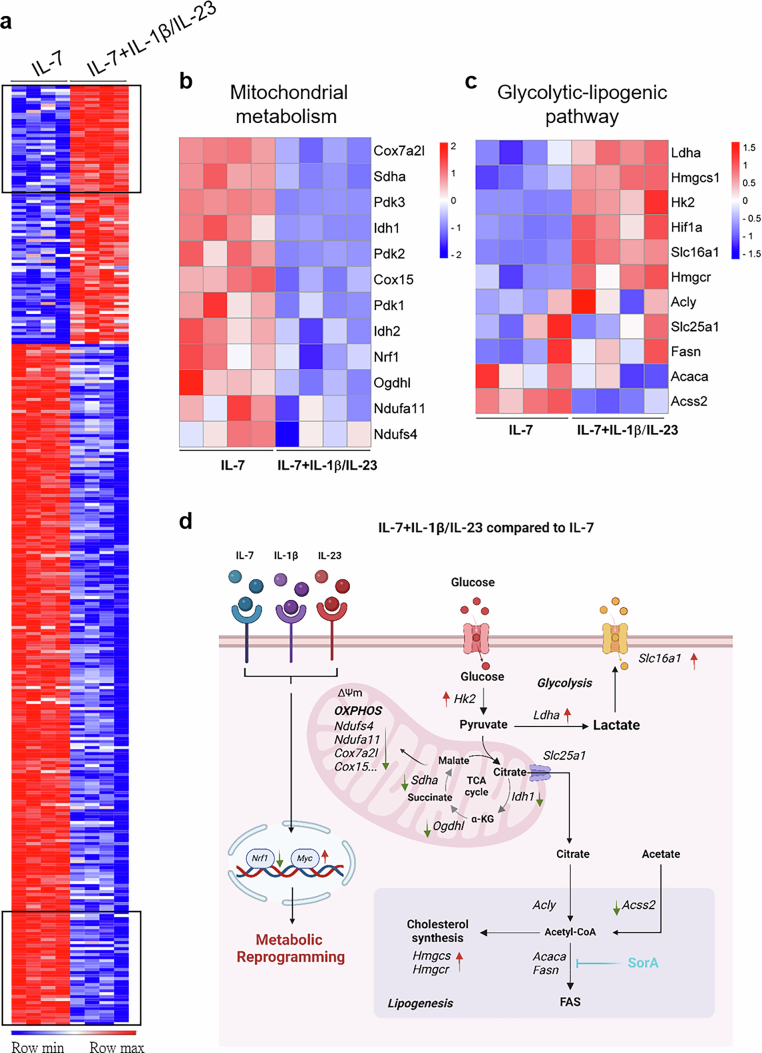
γδT17 cells undergo metabolic reprogramming, reducing mitochondrial metabolism under psoriatic conditions. *In vitro-*expanded γδT17 cells (Lineage^-^γδTCR^+^CD27^-^) were re-seeded on day 9 and stimulated with IL-7 in the presence (psoriatic conditions) or absence (homeostatic conditions) of IL-1β and IL-23 for 3 h. **a**, RNA-seq heatmap showing the upregulated (red) and downregulated (blue) genes ( > 6 fold-change) in γδT17 cells under psoriatic versus homeostatic conditions. The black frame indicates the highest-ranked differentially expressed genes ( > 12 fold-change), listed in Fig. 1a. Data from four independent experiments were collected. For statistical analysis, CLC’s count-based ‘Empirical analysis of Differential Gene Expression’ implementing the ‘Exact Test’ for two-group comparisons and *p* < 0.05 indicates statistically significant differences. **b**, Heatmap of mitochondrial-metabolism-related gene expression. **c**, Heatmap showing the expression of genes associated with the glycolytic-lipogenic pathway. **d**, Schematic model of differentially expressed genes under psoriatic versus homeostatic conditions identified by RNA-seq analysis was created in BioRender. Kao, Y. (2025) https://BioRender.com/x17k361.

To study the metabolic requirements of pathogenic γδT17 cells, we began by differentiating and expanding γδT17 cells using a variation of a previously published protocol^39^. We then cultured the sorted γδT17 cells with IL-7 in the absence or presence of IL-1β and IL-23 to mimic ‘homeostatic’ and ‘psoriatic’ conditions, respectively^24–27^. We performed bulk RNA sequencing (RNA-seq) analysis to identify transcriptional changes that occurred in γδT17 cells on their transition from homeostatic to psoriatic environments (Fig. 1a and Extended Data Fig. 1a). Psoriatic conditions caused γδT17 cells to upregulate cytokine-encoding genes, including *Il17a*, *Il17f* and *Il22* (Fig. 1a). We next explored the functional implications of the upregulated gene signatures by performing pathway analysis using the Molecular Signature Database (MSigDB) hallmark gene set database (Fig. 1b). Multiple highly ranked pathways, including NF-κB, IL-2–STAT5 and IL-6–JAK–STAT3 signalling pathways, were upregulated in γδT17 cells under psoriatic conditions, consistent with previously published reports^40–42^. Unexpectedly, pathways upregulated under psoriatic conditions were related to hypoxia and glycolysis (Fig. 1b). We found that γδT17 cells did indeed downregulate genes related to mitochondrial metabolism and upregulated genes involved in glycolysis under psoriatic conditions (Extended Data Fig. 1b,c). In particular, γδT17 cells downregulated the gene encoding nuclear respiratory factor 1 (*Nrf1*) and components of the mitochondrial respiratory chain (for example, *Sdha* and *Cox7a2l*) and upregulated c-Myc (*Myc*) and hypoxia-inducible factor 1-alpha (*Hif1a*) required to initiate glycolysis^43^ (Extended Data Fig. 1b–d). RNA-seq findings, therefore, revealed that γδT17 cells reprogrammed metabolic pathways in response to psoriatic conditions.

### Reduced mitochondrial activities under psoriatic conditions

Compartment analysis of the RNA-seq data suggested that γδT17 cells expressed higher levels of the FASN complex under psoriatic conditions (Fig. 1c). Therefore, we reasoned that under such conditions, γδT17 cells may engage in aerobic glycolysis to support FAS. Accordingly, we observed that the genes encoding hexokinase 2 (*Hk2*), a lactate dehydrogenase isoform *(ldha)* and a lactate transporter (*Slc16a1*), indicators of an increased glycolytic rate, were highly enriched in γδT17 cells under psoriatic versus homeostatic conditions (Extended Data Fig. 1c,d). The ATP-citrate lyase (encoded by *Acly*) links glucose to lipid metabolism^6,33^ by converting mitochondrial citrate to acetyl-CoA in the cytoplasm for cholesterol metabolism and FAS^24,25,30^. We found that the genes involved in cholesterol metabolism (*Hmgcs1* and *Hmgcr*)^44^ were enriched under psoriatic conditions (Extended Data Fig. 1c). However, the expression pattern of FAS genes (for example, *Fasn* and *Acaca)* was unclear after exposing γδT17 cells to psoriatic conditions for 3 h (Extended Data Fig. 1c). In addition, cytosolic acetyl-CoA can be derived from acetate via acetate-CoA synthetase 2 (ACSS2); nevertheless, γδT17 cells exhibited lower *Acss2* expression under psoriatic than homeostatic conditions (Extended Data Fig. 1c,d).

To verify whether psoriatic conditions reprogrammed γδT17 cells toward aerobic glycolysis, we checked their mitochondrial function by measuring mitochondrial mass and MMP with MitoTracker Green and MitoTracker Red CM-H_2_Xros, respectively (Fig. 1d,e). In agreement with RNA-seq data, γδT17 cells showed reduced mitochondrial activity under psoriatic conditions, as demonstrated by lower mitochondrial mass (Fig. 1d) and MMP (Fig. 1e). We further determined the role of FAS in regulating mitochondrial activity using the ACC inhibitor Soraphen A (SorA). Pre-treating γδT17 cells with SorA before exposure to psoriatic conditions significantly reduced their mitochondrial mass, suggesting that FAS was actively needed to maintain mitochondrial mass under homeostatic conditions (Fig. 1d). Furthermore, SorA treatment showed a tendency to reduce the MMP of γδT17 cells under homeostatic but not psoriatic conditions (Fig. 1e, right). Together, these results imply that although γδT17 cells physiologically rely on FAS-mediated mitochondrial activity, they undergo a metabolic shift under psoriatic conditions.

### The metabolic shift under psoriatic conditions in vivo

**Fig. 2:**
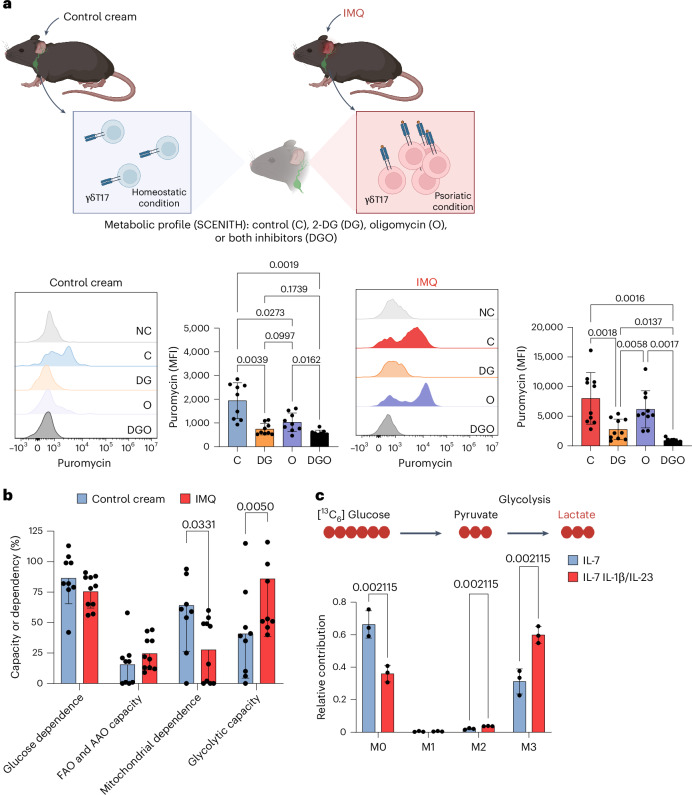
γδT17 cells display distinct metabolic profiles in the mouse model of IMQ-induced psoriasis. The ears of mice were treated topically with control cream or IMQ for five consecutive days. **a**, MFI of puromycin staining was analysed in CD3^+^Vγ4^+^γδT17 cells isolated from the IMQ model using SCENITH^45^ under control conditions and negative control or after the addition of 2-DG, oligomycin or both inhibitors. The illustration was created in BioRender.com. **b**, Percentages of glucose dependence, mitochondrial dependence, glycolytic capacity or fatty acid and amino acid oxidation (FAO and AAO) capacity. **c**, Mass isotopomer distributions of lactate. Data were collected from WT mice treated with control cream (*n* = 9) and IMQ (*n* = 10). In vitro-expanded γδT17 cells were sorted on day 6 and cultured with IL-7 for 3 days. On day 9, cells were re-seeded and stimulated with either IL-7 (homeostatic conditions) or a combination of IL-1β and IL-23 (psoriatic conditions) in the presence of U-[^13^C_6_]-glucose in the last 48 h of the experiment. Pooled means from three independent experiments are shown. Error bars, s.d. *P* values were obtained from one-way ANOVA in **a** and two-way ANOVA in **b** and **c**.

Having shown that pathogenic γδT17 cells rewire metabolic pathways under psoriatic conditions in vitro, we wondered whether the same could be observed in the in vivo IMQ-induced psoriasis mouse model^15^. To this end, we determined the metabolic profiles of γδT17 cells isolated from skin-draining lymph nodes (LNs) of mice treated with either control cream or IMQ (Fig. 2a), by using the Single Cell Metabolism by Profiling Translation Inhibition (SCENITH) protocol^45^. The SCENITH method uses the level of puromycin incorporation to indicate the protein translation rate, which reflects cellular metabolism^45^. Under homeostatic conditions, γδT17 cells of mice treated with control cream were strongly dependent on glucose metabolism and mitochondrial activity, as evidenced by the fact that both 2-deoxy-d-glucose (2-DG) and oligomycin treatment significantly reduced puromycin incorporation compared to the dimethylsulfoxide (DMSO) control (Fig. 2a, left panel). Under psoriatic conditions, however, the oligomycin-treated and DMSO-treated groups showed comparable levels of puromycin incorporation (Fig. 2a, right panel), suggesting that the γδT17 cells from IMQ-treated mice were less dependent on mitochondrial activity than the control mice (Fig. 2a). Following the SCENITH protocol^45^, we calculated the glucose dependence, mitochondrial dependence, glycolytic capacity and fatty acid and amino acid oxidation capacities of γδT17 cells obtained from mice (Fig. 2b). The resulting metabolic profiles indicated that γδT17 cells primarily used glucose rather than fatty acids or amino acids for their energy production under homeostatic and psoriatic conditions (Fig. 2b). Furthermore, γδT17 cells isolated from IMQ-treated mice had a substantially lower mitochondrial dependence and a significantly higher glycolytic capacity than those derived from control mice (Fig. 2b). These results aligned with the data obtained from the γδT17 cells cultured under psoriatic conditions, thus confirming that pathogenic γδT17 cells underwent metabolic reprogramming toward aerobic glycolysis after exposure to inflammatory conditions in vivo.

### Increased glycolytic flux under psoriatic conditions

**Extended Data Fig. 2:**
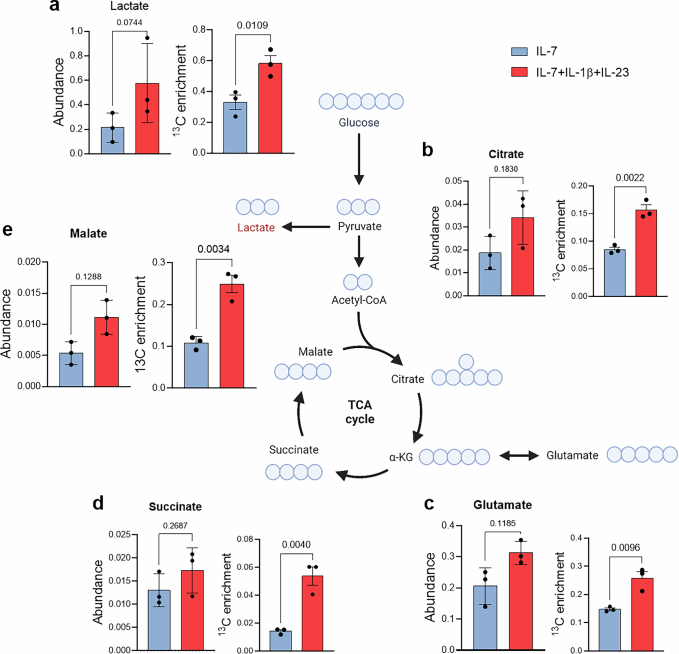
Stable isotope tracing of glucose usage reveals γδT17 cells engage in glycolysis and maintain mitochondrial metabolism under psoriatic conditions. *In vitro*-expanded γδT17 cells were sorted on day 6 and cultured with interleukin (IL)-7 for 3 days. On day 9, cells were re-seeded and stimulated with IL-7 either alone (homeostatic conditions) or combined with IL-1β and IL-23 (psoriatic conditions) for 48 h. The isotopic enrichment of lactate and tricarboxylic acid (TCA) cycle metabolites in γδT17 cells on day 11 was determined by incubating the cells with U-[^13^C_6_]-glucose in the last 48 h of the experiment. Metabolite abundance (left-hand graphs) and fractional glucose contribution analysis (right-hand graphs) revealed the levels of lactate **a** and TCA cycle intermediates and associated metabolites, including citrate **b**, glutamate **c**, succinate **d**, and malate **e** in the γδT17 cells cultured under psoriatic versus homeostatic conditions. Pooled means from three independent experiments are shown. Error bars represent standard deviation (SD), and *p*-values were obtained using a two-sided t test. The graphic was created in BioRender. Kao, Y. (2025) https://BioRender.com/s40a656.

**Extended Data Fig. 3:**
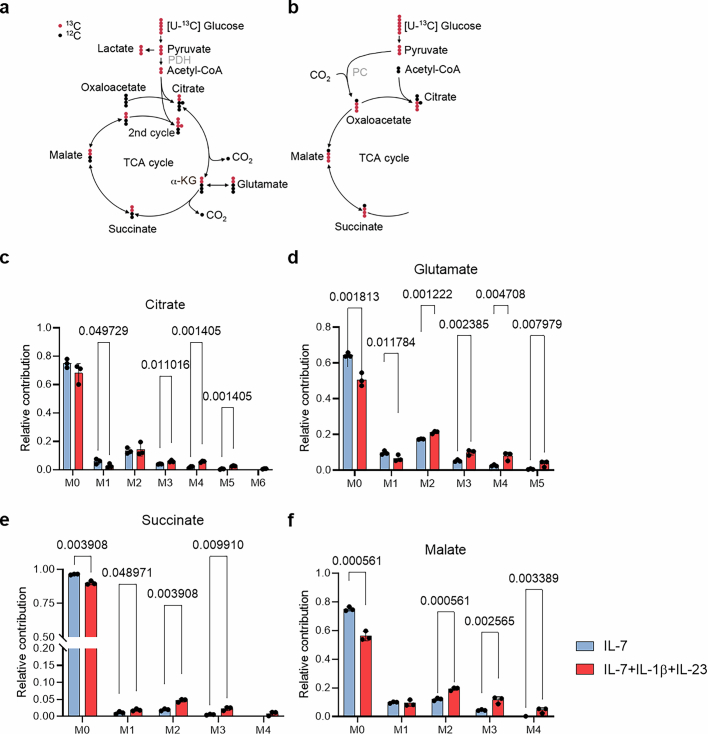
Stable isotope tracing of glucose usage reveals that γδT17 cells maintain mitochondrial metabolism under psoriatic conditions. *In vitro*-expanded γδT17 cells were sorted on day 6 and cultured with IL-7 for 3 days. On day 9, cells were re-seeded and stimulated with either IL-7 (homeostatic conditions) or a combination of IL-1β and IL-23 (psoriatic conditions) for 48 h. **a**, **b**, Schematic representation of U-[^13^C_6_]-glucose labeling of central carbon metabolism and resulting atom transitions. M2 isotopologues of TCA cycle metabolites result from pyruvate dehydrogenase (PDH) flux in **a**, while M3 isotopologues result from pyruvate flux into oxaloacetate through pyruvate carboxylase (PC) in **b**. M2 oxaloacetate condensing with M2 Acetyl-CoA yields M4 isotopologues, which indicate the cycling of TCA cycle metabolites. **c-f**, Mass isotopomer distributions of TCA cycle metabolites, including citrate **c**, glutamate **d**, succinate **e**, and malate **f** in γδT17 cells on day 11 were determined by incubating the cells with U-[^13^C_6_]-glucose in the last 48 h of the experiment. The means of three independent experiments are shown. Error bars represent standard deviation (SD), and *p-*values were obtained using the two-sided t test.

SCENITH analysis indicated predominant glucose usage in γδT17 cells. To elucidate how γδT17 cells use glucose in responding to psoriatic conditions, we coupled our cell culture system with a 48 h U-[^13^C_6_]-glucose-tracing protocol (Fig. 2c and Extended Data Figs. 2 and 3). Mass spectrometry (MS)-based analysis of metabolite abundance revealed that the γδT17 cells increased levels of lactate (Extended Data Fig. 2a and Supplementary Fig. 1) as well as TCA cycle intermediates and related metabolites, including citrate (Extended Data Fig. 2b), glutamate (Extended Data Fig. 2c), succinate (Extended Data Fig. 2d) and malate (Extended Data Fig. 2e). Fractional contribution analysis demonstrated significant enrichment of ^13^C in the products of the glycolytic pathway (Fig. 2c) and TCA cycle intermediates (Extended Data Fig. 2b–e). We found that ~30% of the γδT17 cells cultured under homeostatic conditions contained isotopically enriched lactate (Fig. 2c), and this figure rose to ~60% when γδT17 cells were exposed to psoriatic conditions (Fig. 2c). These findings imply that glycolytic flux was significantly upregulated in γδT17 cells on transitioning from homeostatic to psoriatic conditions. The amount of ^13^C incorporation (Extended Data Fig. 3b) into citrate (Extended Data Fig. 3c), glutamate (Extended Data Fig. 3d), succinate (Extended Data Fig. 3e) and malate (Extended Data Fig. 3f) also increased under psoriatic conditions. Among these, the increase in glucose labelling was particularly prominent for the M2, M3 and M4 isotopomers (Extended Data Fig. 3c–f). Collectively, these data suggest that although γδT17 cells maintained their glucose usage for the TCA cycle under both homeostatic and psoriatic conditions, the psoriatic conditions caused them to further increase their glycolytic flux.

### Enriched proteins for biosynthesis under psoriatic conditions

**Fig. 3:**
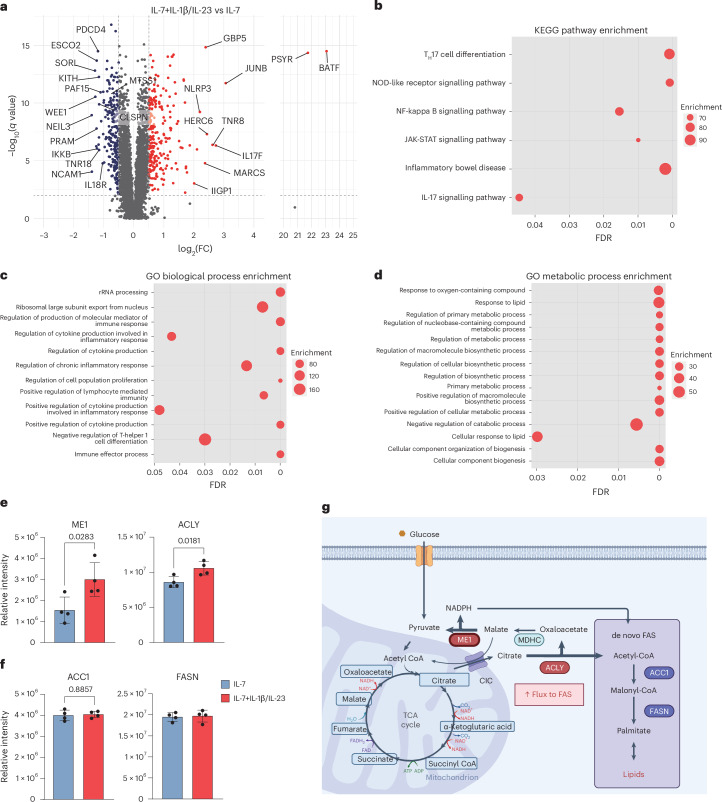
γδT17 cells enrich proteins required for signalling pathways and biosynthetic processes under psoriatic conditions. On day 9, in vitro-expanded γδT17 cells were re-seeded and stimulated with IL-7 alone (homeostatic conditions) or combined with IL-1β and IL-23 (psoriatic conditions) for 24 h before proteomic analysis. **a**, Proteins significantly upregulated or downregulated (log_2_(fold change) > 0.5) under psoriatic versus homeostatic conditions. Data from four independent experiments were collected for the proteomic analysis. *P* values were obtained using the two-sided Benjamini–Hochberg-corrected *t*-test; *P* < 0.01 indicates statistically significant differences. **b**–**d**, KEGG (**b**) and Gene Ontology (GO) pathway enrichment analyses of biological (**c**) and metabolic processes (**d**) with proteins upregulated (red) under psoriatic versus homeostatic conditions. **e**,**f**, The bar graphs show the four proteins involved in glycolytic–lipogenic metabolic processes, including ME1 and ACLY (**e**) and ACC1 and FASN (**f**), which were differentially expressed in γδT17 cells exposed to psoriatic (red) versus homeostatic (blue) conditions. **g**, Schematic model of upregulated proteins under psoriatic versus homeostatic conditions, created in BioRender.com. The mean of technical triplicates from four independent experiments is been shown; error bars, s.d. *P* values were determined using *t*-tests. The enrichment scores and false discovery rate (FDR) were estimated using STRING.

We next investigated potential mechanisms underlying how pathogenic γδT17 cells underwent metabolic reprogramming toward aerobic glycolysis under psoriatic conditions. We performed an MS-based proteomic analysis of γδT17 cells cultured in vitro under psoriatic (IL-7 plus IL-1β/IL-23) or homeostatic (IL-7 alone) conditions. Among the 6,140 proteins identified in γδT17 cells, 237 were significantly upregulated and 213 were significantly downregulated under psoriatic versus homeostatic conditions (Fig. 3a). Strikingly, the expression of basic ATF-like leucine zipper transcription factor (BATF) underwent a log_2_(fold change) > 20 in the γδT17 cells under psoriatic conditions owing to the deficient levels under homeostatic conditions (Fig. 3a). Nevertheless, BATF deficiency in mice did not alter the frequencies of physiological γδ T cell subsets or their IL-17 production^46^. The Kyoto Encyclopedia of Genes and Genomes (KEGG) pathway enrichment analysis showed that under psoriatic conditions, γδT17 cells enriched proteins that were required for T_H_17 cell differentiation and involved in the NF-κB–JAK-STAT–IL-17 signalling pathways (Fig. 3b), as previously described^47^. As expected, Gene Ontology term analysis indicated that the proteins upregulated under psoriatic conditions were involved in translation, effector function and cytokine production (Fig. 3c). Therefore, we speculated that under psoriatic conditions, γδT17 cells reprogrammed their metabolism to support pro-inflammatory effector functions. Indeed, we observed that under psoriatic conditions, γδT17 cells upregulated the metabolic processes required for biosynthesis (Fig. 3d) while downregulating those required for cell division and DNA metabolic processes (Supplementary Fig. 2). Next, we evaluated which individual proteins were involved in the metabolic switch induced by psoriatic inflammation. Interestingly, our proteomic data suggested that γδT17 cells significantly increased the expression of ACLY and malic enzyme 1 (ME1) under psoriatic conditions (Fig. 3e–g) while maintaining comparable levels of ACC1 and FASN (Fig. 3f). ACLY provides cytosolic acetyl-CoA required for FAS by converting citrate from the citrate shuttle system^6,30,33^ (Fig. 3g). As a result, elevated ACLY expression can increase the acetyl-CoA supply for FAS and generate oxaloacetate as a byproduct^33^ (Fig. 3g). Cytoplasmic oxaloacetate is converted to malate and then to pyruvate by ME1 to rejoin the glycolytic pathway^48–50^ (Fig. 3g). ME1, an NADP-dependent enzyme, generates NADPH for FAS^49,50^ (Fig. 3g). Together, these data suggest that psoriatic conditions reprogramme the metabolic pathways of γδT17 cells linking to FAS along with their effector functions.

### Upregulated FAS to support neutral lipid synthesis

**Fig. 4:**
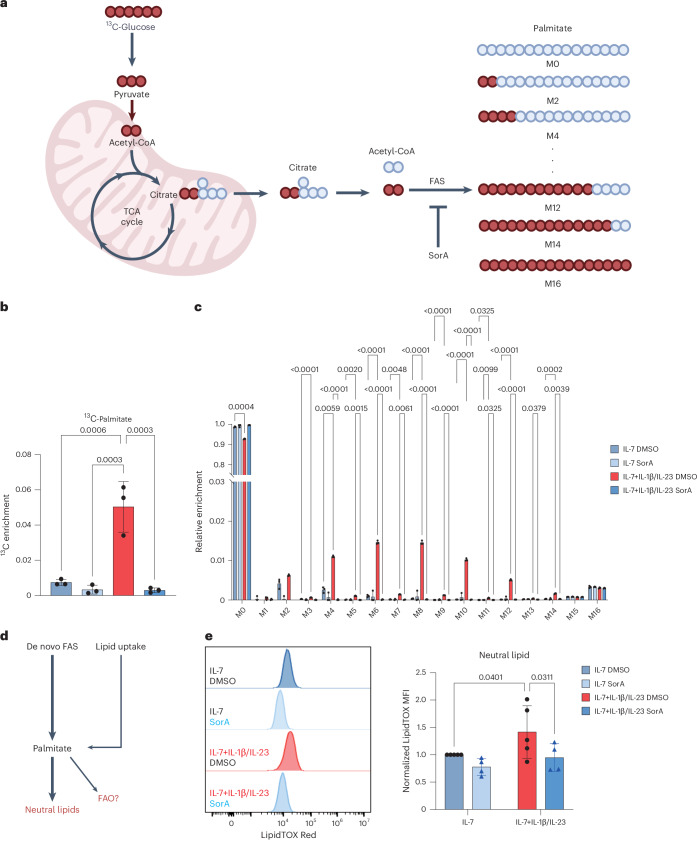
Psoriatic conditions upregulate de novo FAS in γδT17 cells. On day 9, in vitro-expanded γδT17 cells were re-seeded and stimulated with IL-7 alone (homeostatic conditions) or in combination with IL-1β and IL-23 (psoriatic conditions) for 24 h or 48 h in the presence or absence of indicated SorA concentration. **a**, Schematic outcome diagram with stable isotope tracing of U-[^13^C]-glucose into FAS; created in BioRender.com. **b**,**c**, Isotopic palmitate enrichment (**b**) and fractional contribution (**c**) in γδT17 cells were assessed after 48 h of U-[^13^C]-glucose treatment. The means of technical replicates from three independent experiments are shown in **b** and **c**. **d**, Schematic diagram of palmitate usage. **e**, Intracellular neutral lipid content was evaluated by LipidTOX Red. Pooled means of LipidTOX Red MFIs were normalized to those of the IL-7/DMSO condition, obtained from independent experiments of *n* = 5 for DMSO-treated and *n* = 4 for SorA-treated conditions. Error bars, s.d. *P* values were obtained using one-way ANOVA for **b** and the two-way ANOVA for **c** and **e**.

Given that the expressions of two essential components supporting FAS were increased under psoriatic conditions, we reasoned that γδT17 cells altered their glucose metabolism in favour of glycolysis coupling to FAS. To assess whether γδT17 cells used glucose-derived carbon for de novo FAS, we next incubated the cells with U-[^13^C_6_]-glucose and determined the incorporation of ^13^C into palmitate (Fig. 4a). In line with our hypothesis, we observed that γδT17 cells incorporated fivefold more ^13^C into palmitate under psoriatic than under homeostatic conditions (Fig. 4b). We found that the amounts of the even isotopomers (M4–M14) increased under psoriatic versus homeostatic conditions (Fig. 4c), indicating that the contribution of U-[^13^C_6_] glucose to the lipogenic acetyl-CoA pool was increased. Furthermore, SorA treatment completely abolished the isotopic enrichment of palmitate, indicating that this process was ACC-dependent (Fig. 4a–c). Taken together, these findings imply that γδT17 cells significantly increase ACC-mediated FAS under psoriatic conditions.

**Extended Data Fig. 4:**
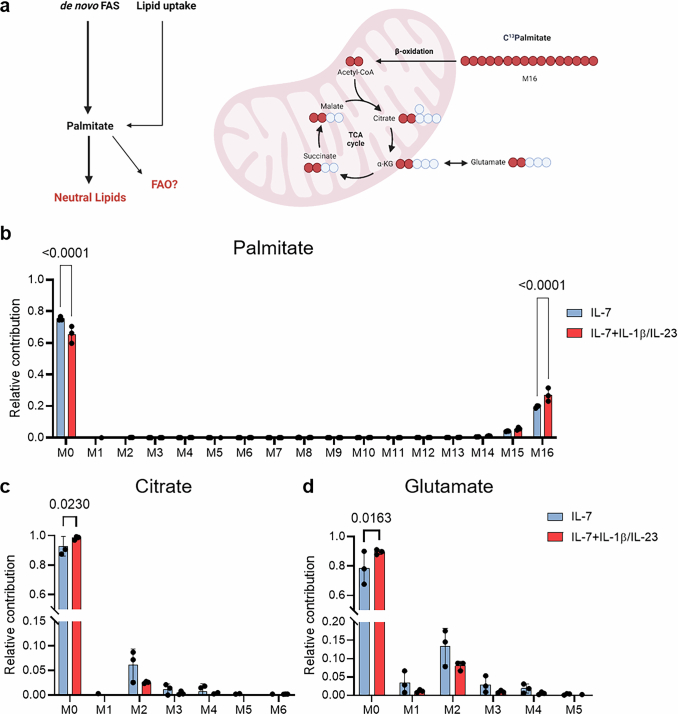
Stable isotope tracing of palmitate usage reveals that γδT17 cells decrease their fatty acid oxidation flux under psoriatic conditions. *In vitro*-expanded γδT17 cells were sorted on day 6 and cultured with IL-7 for 3 days. On day 9, cells were re-seeded and stimulated with either IL-7 (homeostatic conditions) or a combination of IL-1β and IL-23 (psoriatic conditions) for 24 h. **a**, Schematic model of potential palmitate usage was created in BioRender. Kao, Y. (2025) https://BioRender.com/l79h895. **b**, Schematic representation of U-[^13^C_16_]-palmitate labeling of central carbon metabolism into the cycling of TCA cycle metabolites. **c**, **d**, Mass isotopomer distributions of TCA cycle metabolites, including citrate **c** and glutamate **d** in γδT17 cells on day 10 were determined by incubating the cells with U-[^13^C_6_]-glucose in the last 24 h of the experiment. The means of three independent experiments are shown. Error bars represent standard deviation (SD), and *p-*values were obtained using the two-way ANOVA. ns p > 0.05.

To investigate whether γδT17 cells increase FAS to provide palmitate as fuel for fatty acid oxidation, we determined the use of palmitate by stable isotope tracing with U-[^13^C_16_]-palmitate (Extended Data Fig. 4a). We observed that γδT17 cells significantly increased the uptake of U-[^13^C_16_]-palmitate under psoriatic conditions, as indicated by the enrichment of M16 isotopomer (Extended Data Fig. 4b). Strikingly, although fatty acid uptake was increased under psoriatic conditions, we observed reduced rates of β-oxidation, as evidenced by lower abundance of palmitate label in the TCA cycle metabolites, including citrate and glutamate (Extended Data Fig. 4c,d), indicating that most of the increased demand for palmitate input was not used for β-oxidation to generate energy. As excess palmitate can cause lipotoxicity, we speculated that the increased palmitate flux could be actively used as a building block for the synthesis of neutral lipids (Fig. 4d). Therefore, we next checked whether FAS was required for maintaining the intracellular neutral lipid levels in γδT17 cells (Fig. 4e). In line with previous reports^21^, we confirmed that the intracellular neutral lipid content of γδT17 cells increased under psoriatic conditions (Fig. 4e). Importantly, SorA-mediated FAS inhibition prevented the accumulation of these lipids induced by psoriatic conditions (Fig. 4e). Upon psoriatic inflammation, γδT17 cells significantly increased the rate of FAS to provide an increased supply of palmitate to synthesize neutral lipids rather than fuel for fatty acid oxidation.

### γδT17 cells rely on glycolysis to fuel IL-17A production

**Extended Data Fig. 5:**
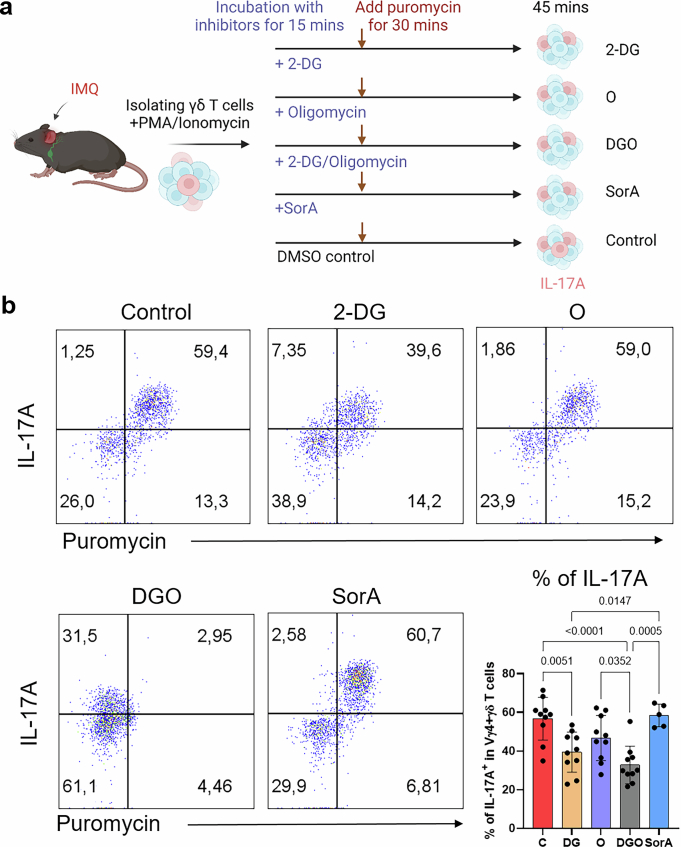
Glycolysis fuels IL-17A production in γδT17 cells from the mouse model of imiquimod-induced psoriasis. **a**, the ears of mice were treated topically with control cream or imiquimod (IMQ) for 5 consecutive days. IL-17A expression was analyzed in CD3^+^Vγ4^+^γδT17 cells isolated from the IMQ model under control conditions (C) and negative control (NC) or after the addition of 2-DG (DG), oligomycin (O), or both inhibitors (DGO), and SorA for 45 minutes (Created in BioRender. Kao, Y. (2025) https://BioRender.com/y77u034) **b**, Representative flow cytometry dot plots and b, percentages showing the IL-17A-expressing γδ T cells obtained under the indicated conditions. Data obtained from 10 mice for the groups of control (C), 2-DG, oligomycin (O), and both inhibitors (DGO), as well as 5 mice for the SorA condition, were shown. Error bars represent standard deviation (SD). *p-*values were obtained using the one-way ANOVA.

Although γδT17 cells rewire their metabolic pathways to engage in aerobic glycolysis and FAS, whether they rely on the glycolytic–lipogenic pathway for IL-17A production under psoriatic conditions is unclear. To this end, we treated γδT17 cells isolated from the IMQ model with 2-DG, oligomycin and SorA, respectively and evaluated which metabolic pathways were required for IL-17A production (Extended Data Fig. 5a,b). We found that the glycolysis inhibitor 2-DG, but not oligomycin or SorA, significantly reduced the ability of γδT17 cells to produce IL-17A (Extended Data Fig. 5a,b). Acute FAS inhibition (45 min) did not affect the IL-17A translation and energetic state of γδT17 cells isolated from the IMQ model, given that puromycin incorporation was comparable between the SorA and DMSO control groups (Extended Data Fig. 5a,b). These results imply that IL-17A-producing γδT17 cells predominately use glycolysis to supply energy for IL-17A production under psoriatic conditions.

### FAS is required for IL-17A expression in γδT17 cells

**Fig. 5:**
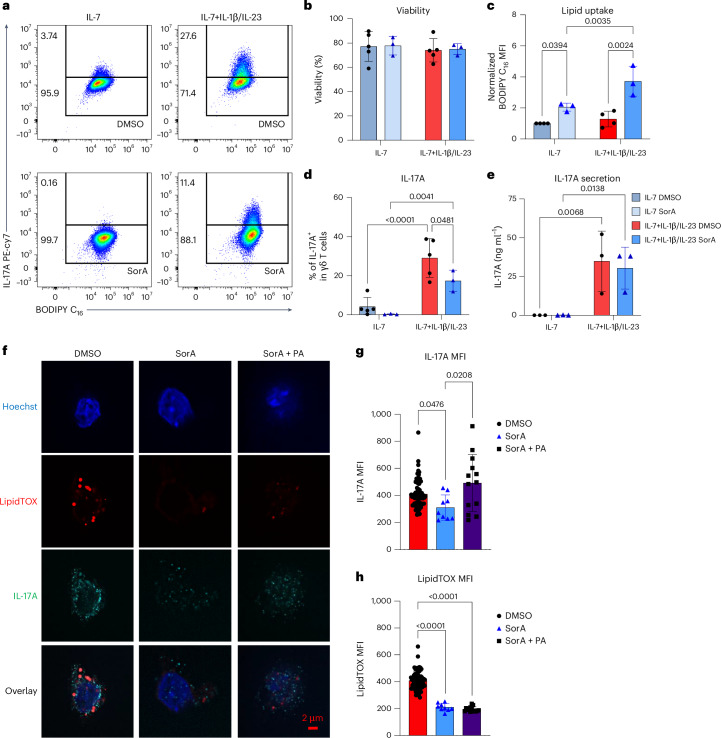
FAS is required for IL-17A expression in γδT17 cells under psoriatic conditions. On day 9, in vitro-expanded γδT17 cells were re-seeded and stimulated with IL-7 alone (homeostatic conditions) or in combination with IL-1β and IL-23 (psoriatic conditions) for 24 h in the presence or absence of 1,000 nM SorA concentration and palmitate (PA) supplementation. **a**, Flow cytometry gating strategy for IL-17A. **b**, Viability of γδT17 cells. **c**, Normalized MFI of lipid uptake measured by BODIPY C_16_. **d**, Percentages of IL-17A^+^ in γδ T cells. **e**, IL-17A secretion in the supernatant of cultured γδT17 cells on day 10 of the indicated conditions. **f**, Representative images of LD and IL-17A expression, evaluated by LipidTOX and anti-IL-17A antibody, respectively. **g**,**h**, MFIs of IL-17A (**g**) and LipidTOX (**h**) were determined in each individual cell under psoriatic conditions (IL-7 + IL-1β/IL-23) for 24 h. Pooled means from independent experiments: *n* = 5 for DMSO and *n* = 3 for SorA-treated conditions in **b** and **d**; *n* = 4 for DMSO and *n* = 3 for SorA-treated conditions in **c**; *n* = 3 in **e**. One set of representative image analysis from three independent experiments is shown in **g** and **h**. Error bars, s.d. *P* values were obtained using two-way ANOVA for **b–e** and the one-way ANOVA for **g** and **h**.

**Extended Data Fig. 6:**
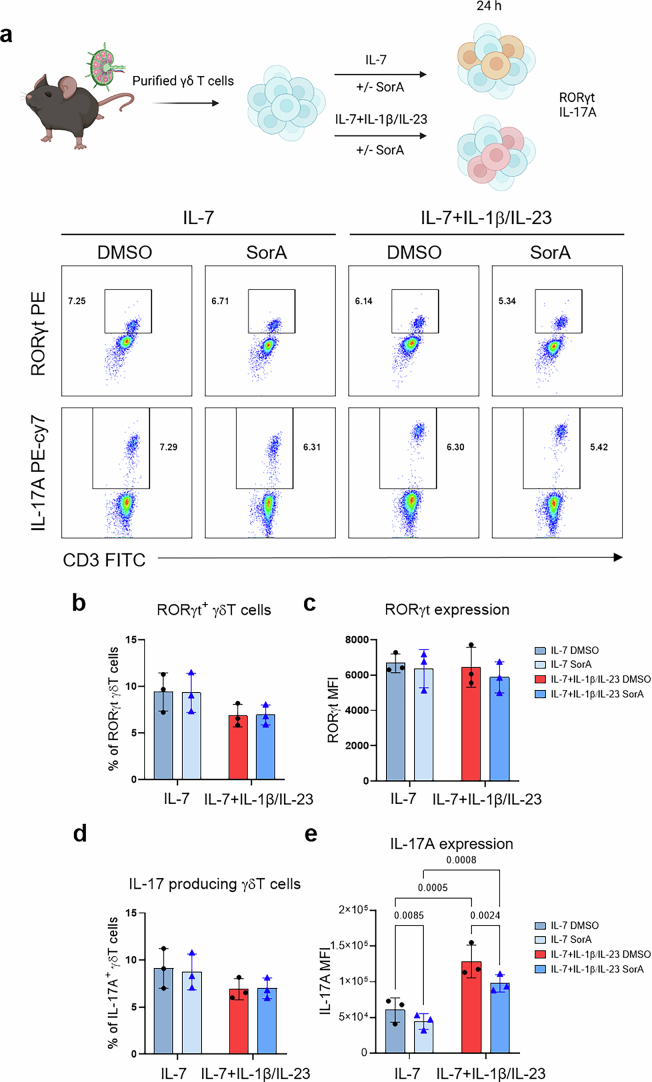
24-h FAS inhibition decreases IL-17A expression in γδT17 cells isolated from LNs without affecting RORγt expression. **a**, Murine γδ T cells isolated from lymphoid organs cultured with IL-7 or IL-7, IL-23, IL-1β for 24 h in the presence or absence of 1000 nM SorA. Cells were re-stimulated with PMA/Ionomycin in the last 4 h (Created in BioRender. Kao, Y. (2025) https://BioRender.com/q70s671). Representative gating strategy of RORγt and IL-17A expression in γδ T cells. **b**, **c**, percentage (**b**) and MFI (**c**) of RORγt within purified γδ T cells. **d**, **e**, percentages (**d**) and MFI (**e**) showing the IL-17A-expressing γδ T cells obtained under the indicated conditions. Pooled means from three independent experiments are shown and error bars represent SD. *p-*values were obtained using the two-way ANOVA.

Having shown that FAS inhibition significantly reduced neutral lipid accumulation induced by psoriatic conditions (Fig. 4e), we were next interested in whether 24 h FAS inhibition could affect IL-17A, given that FAS was required for biosynthetic needs under psoriatic conditions. To this end, we treated γδT17 cells directly isolated from the LNs of WT mice with SorA for 24 h (Extended Data Fig. 6a). After 24 h, approximately 7% of γδT17 cells were maintaining retinoic acid receptor-related orphan receptor-gamma-t (RORγt) and IL-17A expression in response to IL-1β and IL-23 (Extended Data Fig. 6b–e). SorA treatment did not affect the percentage and MFI of RORγt^+^ or the percentage of IL-17A^+^ cells after 24 h exposure to IL-1β and IL-23 (Extended Data Fig. 6b–e). However, 24 h SorA treatment significantly reduced the expression levels of IL-17A, as measured by MFI (Extended Data Fig. 6e), suggesting that inhibiting FAS does not induce new IL-17A producers but is important for the amount of IL-17A produced by γδT17 cells. We further addressed the role of FAS in the last 24 h of the in vitro-differentiated-and-expanded γδT17 cells (Fig. 5a–h) and found that 24 h FAS inhibition did not affect the viability of γδT17 cells under both homeostatic and psoriatic conditions (Fig. 5b). However, SorA-treated γδT17 cells had significantly increased lipid uptake under psoriatic conditions to compensate for FAS inhibition (Fig. 5c), indicating the importance of lipids to γδT17 cells. Exposure to psoriatic conditions caused around 30% of the γδT17 cells to actively produce IL-17A (Fig. 5d). By contrast, 24 h FAS inhibition with SorA significantly reduced IL-17A^+^γδT17 cells to around 16% (Fig. 5d). To assess whether the decreased percentage of IL-17A^+^γδT17 cells was the consequence of enhanced secretion, we further collected the supernatant to determine the levels of secreted IL-17A (Fig. 5e) and found that 24 h FAS inhibition did not affect IL-17A secretion (Fig. 5e). We also examined whether FAS inhibition reduced the percentage of IL-17A^+^γδT17 cells by limiting cell proliferation using the intracellular proliferation marker Ki-67 (Supplementary Fig. 3) and found that 24 h FAS inhibition or lipid-deprived conditions did not affect the percentage and MFI of Ki-67 expression (Supplementary Fig. 3a,b), indicating that neither 24 h FAS inhibition nor lipid-deprived conditions impaired the proliferation of γδT17 cells. These data suggest that 24 h FAS inhibition decreased the percentage of IL-17A^+^γδT17 cells by limiting their IL-17A expression.

**Extended Data Fig. 7:**
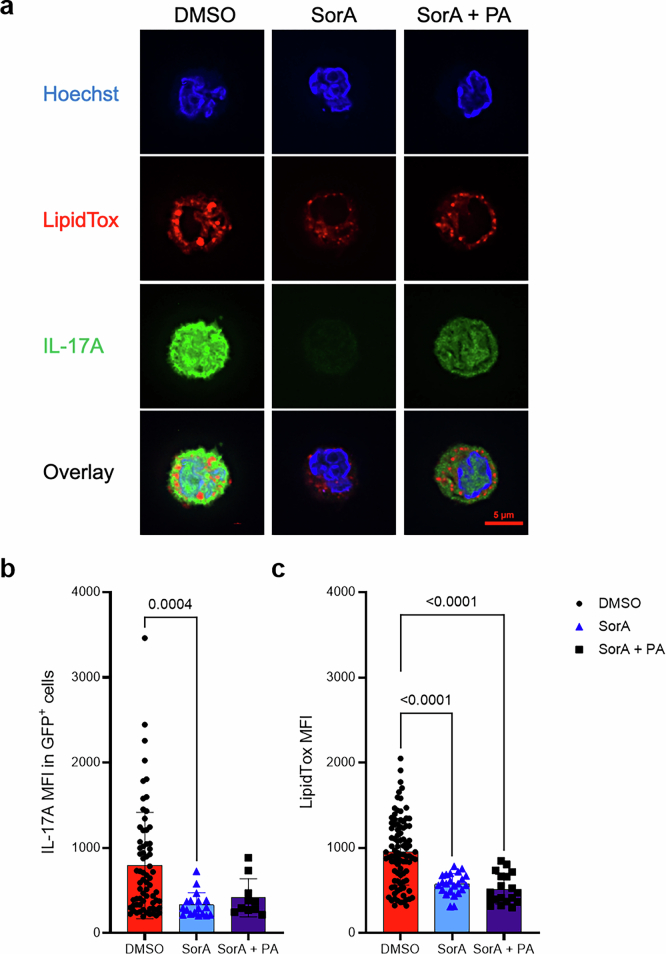
24 h-FAS inhibition limits IL-17A and LipidTOX levels in the *in vitro* culture of γδT17 cells from IL-17A reporter mice. *In vitro*-expanded γδT17 cells from IL-17A-GFP-reporter (C57BL/6-Il17atm1Bcgen/J) mice were sorted on day 6 and cultured with IL-7 for 3 days. On day 9, cells were re-seeded and stimulated with either IL-7 (homeostatic conditions) or a combination of IL-1β and IL-23 (psoriatic conditions) for 24 h. **a**, Representative immunofluorescence images of Lipid droplets (LipidTOX, red), IL-17A-GFP (green), and nuclei (Hoechst 33342, blue) by confocal microscopy. Scale bars represent 5 μm. **b**, mean fluorescence intensities (MFIs) of IL-17A-GFP were shown. **c**, MFIs of LipidTOX were shown. The means of pooled data from 2 mice are shown. At least 10 cells were shown in each condition from one set of the independent experiment. Error bars represent standard deviation (SD), and *p-*values were obtained using the one-way ANOVA.

γδT17 cells were shown to have a higher lipid droplet (LD) content than γδIFN cells under homeostatic conditions^21^. Therefore, we next checked whether FAS-derived palmitate was required for maintaining the intracellular neutral lipid levels and IL-17A expression in γδT17 cells, using confocal microscopy. In line with previous reports^21^, we confirmed that γδT17 cells were loaded with LDs under psoriatic conditions and that the accumulation of these lipids was abrogated after 24 h of SorA-mediated FAS inhibition (Fig. 5f). IL-17A showed a punctate pattern dispersed throughout the cytoplasm in γδT17 cells under psoriatic conditions (Fig. 5f). Furthermore, IL-17A MFI was significantly reduced in the γδT17 cells after 24 h of FAS inhibition (Fig. 5g). Palmitate supplementation restored the IL-17A expression in the SorA-treated γδT17 cells, suggesting that FAS-derived palmitate is required for IL-17A production (Fig. 5g). Interestingly, palmitate supplementation did not replenish the intracellular neutral lipid content of SorA-treated γδT17 cells to control levels, indicating that either γδT17 cells were rapidly consuming palmitate or exogenous palmitate did not favour lipid storage as FAS (Fig. 5h). Furthermore, palmitate supplementation rescued IL-17A expression in the SorA-treated γδT17 cells without replenishing LD accumulation and LipidTOX MFI to the same levels as the DMSO control, suggesting that IL-17A expression is independent of the presence of LDs (Fig. 5f–h). We further confirmed our findings by using IL-17A-GFP-reporter (C57BL/6-*Il17a*^*tm1Bcgen*^/J (IL17A-IRES-GFP-KI) mice to culture γδT17 cells following the same in vitro-differentiated-and-expanded protocol (Extended Data Fig. 7a–c). Similarly, SorA significantly reduced IL-17A–GFP expression levels of γδT17 cells from IL-17A-GFP-reporter mice but not in the presence of additional palmitate supplement (Extended Data Fig. 7b). These results suggest that the γδT17 cells are strongly dependent on FAS to supply palmitate and support their IL-17A production under psoriatic conditions.

**Extended Data Fig. 8:**
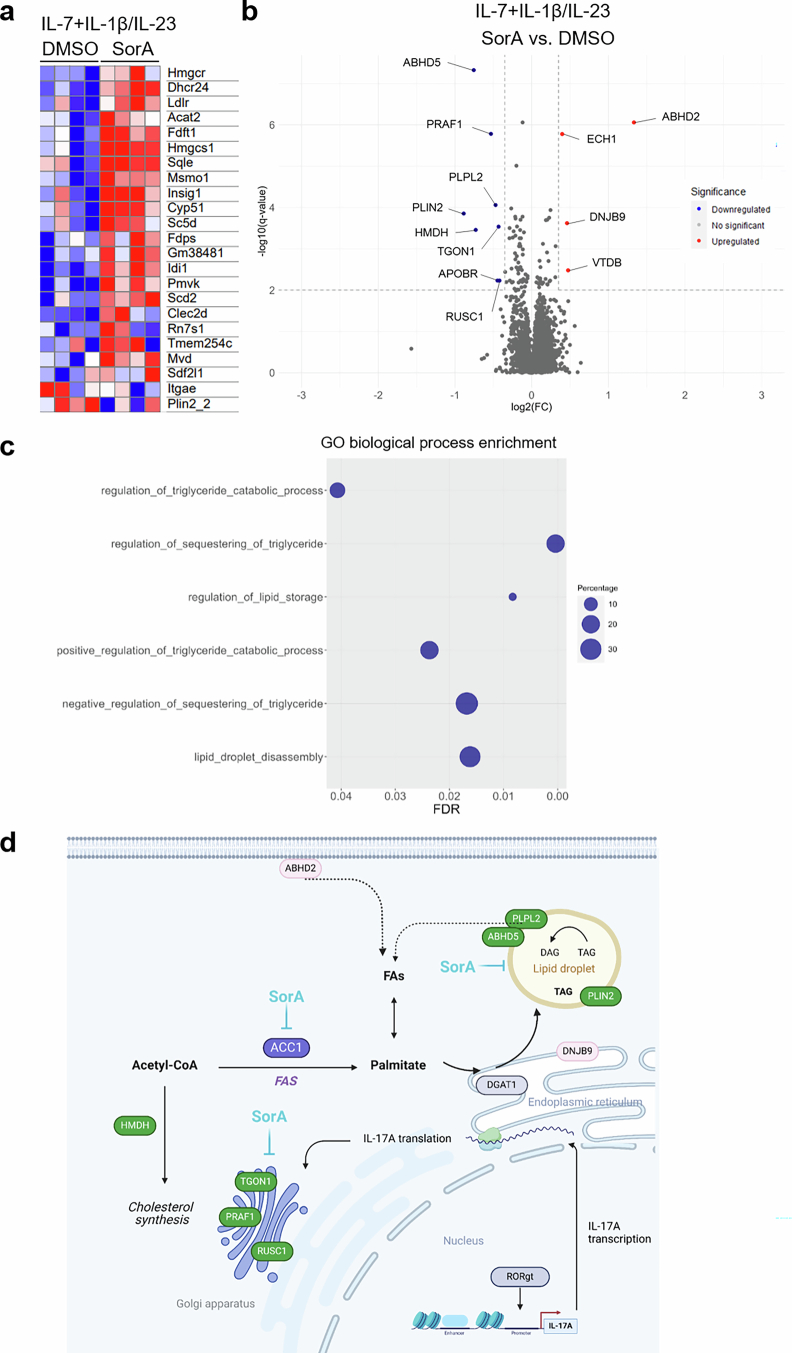
RNA-seq and proteomic analysis demonstrate that γδT17 cells downregulated lipid storage upon FAS inhibition *in vitro.* *In vitro-*expanded γδT17 cells (Lineage^-^γδTCR^+^CD27^-^) were sorted on day 6 and cultured with IL-7 for 3 days. On day 9, cells were re-seeded and stimulated with IL-7, IL-1β, and IL-23 (psoriatic conditions) in the presence or absence of the indicated concentration of SorA for 3 h (for RNA-seq analysis) or 24 h (for proteomic analysis); γδT17 cells treated with SorA or DMSO (control) were compared. **a**, Heatmap of genes upregulated (red) and downregulated (blue) in bulk RNA-seq analysis of γδT17 cells in the presence of SorA or DMSO under psoriatic conditions. **b**, Significantly upregulated (red) and downregulated (blue) proteins with >0.35 log2 fold-change (FC) values were identified in the proteomic analysis comparing γδT17 cells treated with SorA and DMSO. **c**, Gene ontology (GO) enrichment analysis shows the biological processes and pathways associated with the downregulated proteins, as shown in **b**. The proteomic analysis results were obtained from four independent experiments, and *p-*values were generated using the two-sided Benjamini-Hochberg corrected t-test. *p* < 0.05 indicates statistically significant differences. **d**, Schematic model of the pathway analysis with downregulated proteins comparing SorA versus DMSO control under psoriatic conditions was created in BioRender. Kao, Y. (2025) https://BioRender.com/x09m732.

To gain more insight into how γδT17 cells adjusted their lipid demand upon FAS inhibition, we performed RNA-seq analysis to characterize γδT17 cells treated with either SorA or DMSO under psoriatic conditions for 3 h (Extended Data Fig. 8a). We observed that γδT17 cells upregulated several genes involved in cholesterol metabolism (for example, *Hmgcr*, *Ldlr*, *Acat2* and *Hmgcs1*) in the first 3 h following FAS inhibition (Extended Data Fig. 8a). We then conducted a proteomic analysis to evaluate the molecular alterations occurring in γδT17 cells at 24 h after FAS inhibition (Extended Data Fig. 8b). Among the 6,140 proteins identified in γδT17 cells after SorA-mediated FAS inhibition, four proteins were overexpressed and eight were underexpressed (Extended Data Fig. 8b). This finding suggests that upon FAS inhibition, γδT17 cells can cope with their cellular lipid demand of FAS by relying on this small set of proteins (Extended Data Fig. 8b). Abhydrolase domain containing 2 (ABHD2) was identified as the highest-ranking upregulated protein (Extended Data Fig. 8b). ABHD2 catalyses the hydrolysis of endocannabinoid arachidonoylglycerol^51^ and serves as a triacylglycerol lipase to release free fatty acids^52^, which may compensate for the reduced lipids generated by FAS under psoriatic conditions (Fig. 4e). The protein expression of the rate-limiting enzyme in cholesterol synthesis, 3-hydroxy-3-methylglutaryl-coenzyme-A reductase (HMDH, encoded by *Hmgcr*), was downregulated after 24 h FAS inhibition (Extended Data Fig. 8b). Gene Ontology pathway analysis of proteomic data indicated that the triglyceride metabolic process and storage were markedly downregulated following 24 h FAS inhibition (Extended Data Fig. 8c). The top-ranking downregulated proteins included three LD-binding proteins, namely lysophosphatidic acid acyltransferase (ABHD5), adipose triglyceride lipase (ATGL/PLPL2) and perilipin 2 (PLIN2) (Extended Data Fig. 8b). ABHD5 interacts with ATGL, which governs the hydrolysis of intracellular triglyceride to release fatty acids from LDs^53,54^. As PLIN2 is susceptible to ubiquitin-mediated proteasomal degradation without intracellular lipids^55^, its expression mirrors the levels of intracellular LDs. In concordance with the LipidTOX staining (Figs. 4e and 5f), SorA treatment reduced PLIN2 expression in γδT17 cells, indicating that intracellular lipid stores were diminished (Extended Data Fig. 8b). Overall, these data suggest that under psoriatic conditions, γδT17 cells use FAS to meet their lipid demands and that FAS inhibition triggers compensatory fatty acid release from LDs, resulting in significantly reduced lipid storage after 24 h. In addition, we found that 24 h FAS inhibition decreased the expression of proteins (prenylated Rab acceptor protein 1 (PRAF1), trans-Golgi network integral membrane protein 1 (TGON1) and AP-4 complex accessory subunit (RUSC1)) located in the Golgi apparatus required for trans-Golgi network and vesicular trafficking, which are essential processes to deliver cytokines^56^ (Extended Data Fig. 8b). Lipid homeostasis has a critical role in Golgi secretory function, as the unique lipid composition of the Golgi membrane is essential for maintaining the trafficking of proteins and lipids^56^. FAS inhibition disrupting lipid balance could significantly impair the efficiency of Golgi secretory function, as suggested by decreased expression of PRAF1, TGON1 and RUSC1. Accordingly, 24 h FAS inhibition prevented γδT17 cells from replenishing their lipid stores under psoriatic conditions, limited palmitate supply and decreased trans-Golgi network, impairing IL-17A production (Extended Data Fig. 8d).

### ACC1 is indispensable for the IL-17A-producing γδT17 cells

**Fig. 6:**
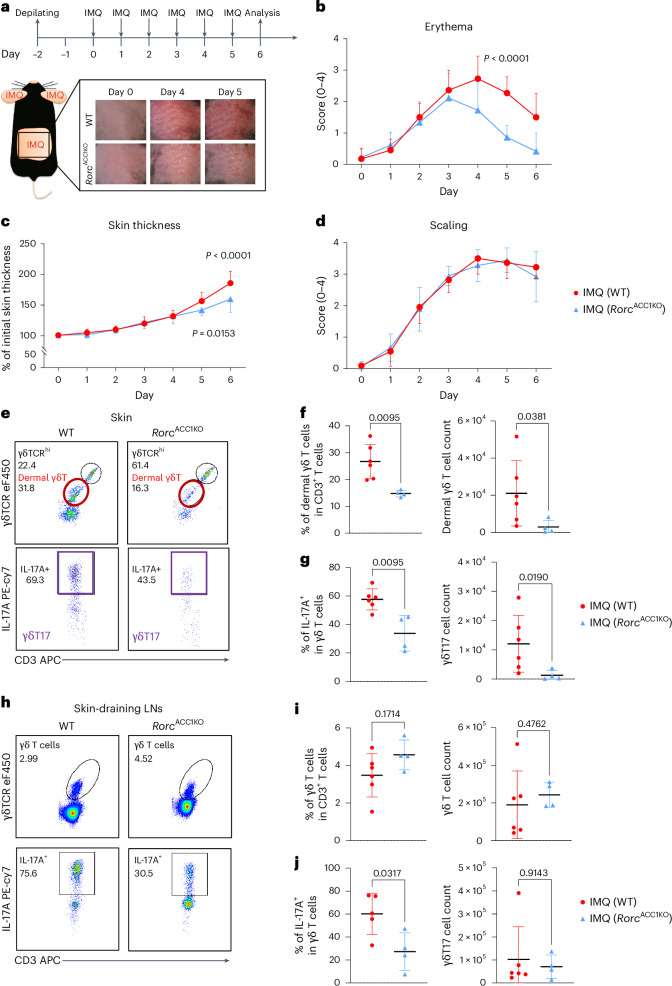
Genetic ablation of ACC1 in RORγt^+^ γδ T cells attenuates IMQ-induced skin inflammation. **a**, Ears and back skin of WT or *Rorc*^ACC1KO^ model mice were treated topically with IMQ for six consecutive days. **b**–**d**, The clinical scores of erythema (**b**), thickness of ear skin (**c**) and clinical scores of scaling (**d**) were measured daily during the IMQ treatment period. **e**, Flow cytometry gating strategy for identifying γδT17 cells (CD3^+^γδTCR^int^IL-17A^+^) within skin samples of the treated ear areas on day 6. **f**,**g**, Percentages and cell numbers of dermal γδ T cells (CD3^+^γδTCR^int^) (**f**) and γδT17 cells (CD3^+^γδTCR^int^IL-17A^+^ (**g**) in the ear skin of WT or *Rorc*^ACC1KO^ mice. **h**, Flow cytometry gating strategy for identifying γδ T cells and IL-17A-producing cells within skin-draining LNs on day 6. **i**,**j**, Percentages and cell numbers of γδ T cells (CD3^+^γδTCR^+^) (**i**) and γδT17 cells (CD3^+^γδTCR^+^IL-17A^+^) (**j**) in the skin-draining LNs of WT or *Rorc*^ACC1KO^ mice. The means of pooled data from three independent experiments are shown. Error bars, s.d. *P* values were determined using two-way ANOVA to compare IMQ-treated WT and *Rorc*^ACC1KO^ mice in **b**–**d**; a two-sided *t*-test was used in **e**–**j**.

Having highlighted the reliance of γδT17 cells on FAS under psoriatic conditions in vitro, we next investigated whether targeting ACC1-mediated FAS in γδT17 cells could attenuate their ability to produce IL-17A and reduce psoriatic inflammation in vivo. We used *Rorc*^ACC1KO^ mice carrying ACC1-deficient γδ T cells^35,41,57^. We exposed the mice to IMQ and monitored the effect of ACC1 deficiency in γδT17 cells on the development of psoriatic inflammation (Fig. 6a). We found that IMQ-treated *Rorc*^ACC1KO^ mice showed significantly reduced erythema on day 5 compared to the IMQ-treated WT mice (Fig. 6b). Furthermore, WT mice developed thicker skin lesions following IMQ exposure than *Rorc*^ACC1KO^ mice (Fig. 6c). ACC1 depletion did not affect skin scaling (Fig. 6d). RORγt is an essential transcription factor for thymic T cell development during the double-positive (CD4^+^CD8^+^) thymocyte phase^57^. Thus, *Rorc*^Cre^ could affect both αβ T cells and γδ T cells^41,57^. These results obtained from the *Rorc*^ACC1KO^ mice imply that targeting ACC1 in αβ T cells and γδT17 cells reduced the extent of psoriatic inflammation in vivo.

**Extended Data Fig. 9:**
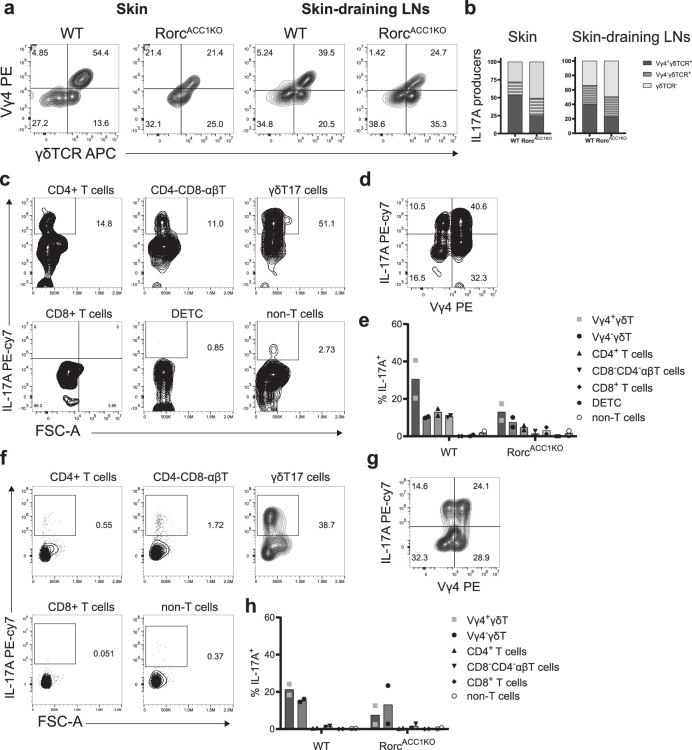
Genetic ablation of ACC1 in RORγt^+^ T cells limits IL-17A production mainly from the Vγ4^+^γδ T cells in the IMQ model. The ears and back skin of wild-type (WT) or Rorc^ACC1KO^ model mice were treated topically with imiquimod (IMQ) for six consecutive days. **a**, Flow cytometry gating strategy for identifying IL-17A-producing cells within skin samples of the treated ear areas and skin-draining LNs on day 6. **b**, Percentages of Vγ4^+^γδ T cells (γδTCR^+^ Vγ4^+^), Vγ4^-^γδ T cells (γδTCR^+^ Vγ4^-^), and non- γδ T cells in the ear skin and skin-draining LNs of WT or Rorc^ACC1KO^ mice (gated on IL-17A^+^ cells). **c**, Flow cytometry gating strategy for analyzing IL-17A+ in CD4^+^ T cells (CD45^+^αβTCR^+^CD4^+^γδTCR^-^), CD8^+^ T cells (CD45^+^αβTCR^+^CD8^+^γδTCR^-^), double-negative αβ T cells (CD45^+^αβTCR^+^CD4^-^CD8^-^γδTCR^-^), DETC (CD45^+^γδTCR^high^), γδT17 cells (CD45^+^γδTCR^int^) and non-T cells within treated-skin. **d**, Flow cytometry gating strategy for IL-17A^+^Vγ4^+^γδ T cells and IL-17A^+^Vγ4^-^γδ T cells from the treated skin. **e**, Percentages of IL-17A in indicated cell types in the treated skin. **f**, Flow cytometry gating strategy for analyzing IL-17A+ in CD4^+^ T cells (CD45^+^αβTCR^+^CD4^+^γδTCR^-^), CD8^+^ T cells (CD45^+^αβTCR^+^CD8^+^γδTCR^-^), double-negative αβ T cells (CD45^+^αβTCR^+^CD4^-^CD8^-^γδTCR^-^), γδT17 cells (CD45^+^γδTCR^int^) and non-T cells in the skin-draining lymph node (LN) on day 6. **g**, Flow cytometry gating strategy for IL-17A^+^Vγ4^+^γδ T cells and IL-17A^+^Vγ4^-^γδ T cells from the skin-draining LNs. **h**, Percentages of IL-17A in indicated cell types in the skin-draining LNs. The data were from WT (n = 2) or Rorc^ACC1KO^ mice (n = 2).

γδT17 cells migrated from the skin-draining LNs into the skin on day 5 after IMQ treatment^18^. Given that ACC1 deletion reduced skin thickening and erythema in IMQ-treated *Rorc*^ACC1KO^ mice on day 5, we next asked whether ACC1 deficiency inhibited γδT17 cell infiltration into the skin, thus attenuating skin inflammation. To this end, we extracted immune cells from the IMQ-treated skin of model mice and characterized the γδ T cell populations by flow cytometry^23^. We observed that IMQ treatment induced the expansion of dermal γδT17 cells (CD3^+^γδTCR^int^; Fig. 6e), which accounted for 20–35% of the total skin T cells on day 6 after IMQ treatment (Fig. 6f). Frequencies and cell numbers of dermal γδT17 cells (CD3^+^γδTCR^in^^t^) were significantly lower in the *Rorc*^ACC1KO^ than WT mice following IMQ treatment (Fig. 6e,f). We also found that ACC1 deficiency further impaired the ability of the γδT17 cells to produce IL-17A in the skin, as evidenced by the lower frequencies of IL-17A^+^dermal γδ T cells in the IMQ-treated *Rorc*^ACC1KO^ than WT mice (Fig. 6g). γδT17 cells accounted for more than 70% of IL-17A-producing cells in the IMQ-treated skin and 60% in the skin-draining LNs of WT mice on day 6 (Extended Data Fig. 9a,b). Their contribution was reduced to below 50% in the IMQ-treated skin of *Rorc*^ACC1KO^ mice (Extended Data Fig. 9a,b). We further characterized IL-17A production in Vγ4^+^γδ T cells (CD45^+^γδTCR^+^Vγ4^+^), Vγ4^−^γδ T cells (CD45^+^γδTCR^+^Vγ4^−^), CD4^+^ T cells (CD45^+^αβTCR^+^CD4^+^γδTCR^−^), CD8^+^ T cells (CD45^+^αβTCR^+^CD8^+^γδTCR^−^), double-negative αβ T cells (CD45^+^αβTCR^+^CD4^−^CD8^−^γδTCR^−^), dendritic epidermal T cells (CD45^+^γδTCR^high^) and non-T cells within treated skin (Extended Data Fig. 9c,d). We observed that Vγ4^+^γδ T cells are the main IL-17A producers, followed by Vγ4^−^γδ T cells (γδTCR^+^ Vγ4^−^) and CD4^+^ T cells (CD45^+^αβTCR^+^CD4^+^γδTCR^−^). *Rorc*^Cre^-mediated ACC1 deficiency reduced IL-17A production in Vγ4^+^γδ T cells and CD4^+^ T cells (Extended Data Fig. 9c–e).

We next assessed whether ACC1 was required for γδT17 cell expansion in the skin-draining LNs and found comparable frequencies and numbers of total γδ T cells between IMQ-treated *Rorc*^ACC1KO^ and WT mice (Fig. 6h,i), suggesting that ACC1 deficiency did not affect γδ T cell expansion. Notably, IMQ-treated *Rorc*^ACC1KO^ mice had significantly lower frequencies of IL-17A^+^γδT17 cells in the skin-draining LNs than the IMQ-treated WT mice, which led to reduced γδT17 cell infiltration into the skin (Fig. 6g–j). Among IL-17A-producing cells on day 6, ACC1 deficiency reduced IL-17A production most in Vγ4^+^γδ T cells (Extended Data Fig. 9f–h). These data highlight the selective role of ACC1 in IL-17A production by γδT17 cells on day 6 (Fig. 6g,j). We further address the effect of ACC1 deficiency on the expression of RORγt. Mice bearing ACC1-deficient γδ T cells showed comparable expression of RORγt^+^ cells in the skin-draining LNs and skin compared to WT mice (Supplementary Fig. 4a,b). We evaluated whether ACC1-deficient γδ T cells engaged any compensatory lipid uptake under psoriatic inflammation in vivo. ACC1-deficient γδT17 cells (RORγt^+^γδTCR^+^) significantly upregulated their lipid uptake capacity but not ACC1-deficient RORγt^−^γδ T cells (RORγt^−^γδTCR^+^) in the skin-draining LNs compared to their WT counterparts (Supplementary Fig. 4a). As a result, ACC1-deficient γδT17 cells exhibited intracellular lipid levels comparable to WT (Supplementary Fig. 4a). Interestingly, ACC1-deficient γδT17 cells in the skin did not increase lipid uptake capacity, suggesting that these γδT17 cells could have a lower dependency on ACC1-mediated FAS compared to those ACC1-deficient γδT17 cells in the draining LNs (Supplementary Fig. 4a,b). Nevertheless, the lipid availability in physiological conditions and potentially different intracellular lipid distributions between endogenous and exogenous lipid sources meant that ACC1-deficient γδT17 cells failed to meet their lipid demand for IL-17A production in vivo (Fig. 6g,j). In summary, ACC1 deficiency restrains IL-17A-producing γδT17 cells in the skin and skin-draining LNs of mice with IMQ-induced psoriasis.

**Fig. 7:**
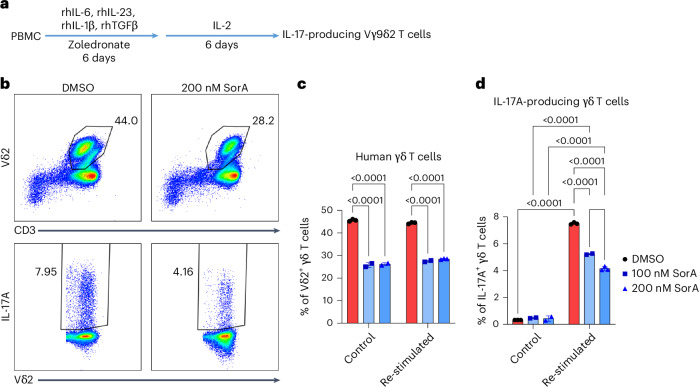
SorA treatment reduces the frequencies of human IL-17A-producing Vδ2^+^γδ T cells. **a**, Human peripheral blood mononuclear cells (PBMCs) were stimulated with zoledronate, rhIL-6, rhIL-23, rhIL-1β and rhTGF-β for 6 days. On day 6, half of the old media was removed and replaced with fresh media containing IL-2 in the presence or absence of the indicated concentration of SorA for another 6 days^58^. **b**, Flow cytometry gating strategy of total and IL-17A-producing Vδ2^+^γδ T cells. **c**,**d**, The cells were re-stimulated with PMA/ionomycin for 4 h in the presence of BFA for the last 2 h; percentages of Vδ2^+^γδ T cells (CD3^+^Vδ2^+^) (**c**) and IL-17A^+^Vδ2^+^γδ T cells (CD3^+^Vδ2^+^IL-17A^+^) (**d**) are shown. The means of one representative set of three independent experiments are shown. Error bars, s.d. *P* values were determined using two-way ANOVA.

Finally, we extended our findings to in vitro-expanded human IL-17A-producing Vδ2^+^γδ T cells^58^ (Fig. 7a). Human γδ T cells can be divided into three subtypes: Vδ1, Vδ2 and Vδ3, based on δ chain usage. However, the link between γδTCR usage and IL-17A-producing capacity is still controversial^58,59^. Recent evidence suggested that a committed Vγ9Vδ2 γδ T cell subset derived from adult blood was biased toward the γδT17 cell profile^59^. Therefore, we optimized a protocol using peripheral blood to have maximum IL-17A^+^Vδ2 T cells^58^ to study human γδT17 cells (Fig. 7). We found that FAS inhibition attenuated human Vδ2^+^γδ T cell expansion and their IL-17A production (Fig. 7b–d). These results suggest that ACC1-mediated FAS is indispensable for the IL-17A-producing function of pathogenic γδT17 cells under psoriatic conditions in mice and humans.

## Discussion

Metabolic dysregulation is associated with various immune-mediated inflammatory conditions, including type 2 diabetes, nonalcoholic fatty liver disease (NAFLD), atherosclerosis and psoriasis^60,61^. Although a previous publication has highlighted the metabolic dichotomy of γδ T cell subsets^21^, little is known about the metabolic profiles of γδT17 cells under pathogenic conditions. Here, we found that γδT17 cells responded to the pro-inflammatory cytokines IL-1β and IL-23, hallmarks of psoriatic inflammation, by undergoing metabolic reprogramming. Mitochondrial metabolism is required during γδT17 cell differentiation in the thymus and is maintained as the γδT17 cells exit the thymus and infiltrate peripheral tissues and even tumours^21^. Unexpectedly, we showed that under psoriatic conditions, γδT17 cells underwent metabolic reprogramming in favour of aerobic glycolysis, both in vitro (exposure to IL-1β and IL-23) and in vivo (IMQ-treated mice), which was essential for their IL-17A production. This metabolic reprogramming, the so-called Warburg effect, was identified using the SCENITH protocol^45^ of pathogenic γδT17 cells isolated from the IMQ-treated mice and may provide a potential therapeutic target. In support of this idea, we showed that 2-DG inhibition effectively blocked IL-17A production by γδT17 cells under psoriatic conditions. Notably, the pro-inflammatory effects mediated by IL-1β and IL-23 exhibit a broader pathophysiological relevance in addition to psoriatic disease models. Therefore, the metabolic reprogramming in response to IL-1β and IL-23 and the proteomic database of γδT17 cells in the current study provide insights for other inflammatory models, such as neuroinflammation^18^.

Naive αβ T cells rewire their metabolism to fulfil their differentiation requirements, which is initiated by mTOR signalling upon αβ TCR stimulation^29,62–65^. Similarly, IL-1β and IL-23 activate the mTOR pathway in γδT17 cells^25^, which could trigger metabolic reprogramming to support their IL-17 production under pathogenic conditions. In agreement with these data, we demonstrated that γδT17 cells adopted aerobic glycolysis as their primary energy source to support their IL-17 production. RNA-seq data indicated that this metabolic reprogramming of γδT17 cells was associated with *Myc* upregulation. Interestingly, a similar metabolic shift from oxidative phosphorylation (OXPHOS) to glycolysis occurs during thymic development, when γδ T cell progenitors commit to becoming IFNγ-producing γδ T cells^21^. We speculate that this metabolic shift triggered by psoriatic conditions may blur the distinction between IL-17-producing and IFNγ-producing γδ T cell populations. Indeed, our RNA-seq data suggested that γδT17 cells upregulated both IL-17A and IFNγ mRNA transcripts in response to IL-1β and IL-23. Nevertheless, whether these γδT17 cells co-expressed IL-17A and IFNγ remains to be investigated.

γδT17 cells exhibit more active mitochondrial metabolism than γδIFN cells^21^. Although γδT17 cells were forced to switch toward aerobic glycolysis under psoriatic conditions, γδT17 cells maintained a high mitochondrial metabolic rate even under psoriatic conditions, as indicated by the active isotope labelling of TCA cycle metabolites during culture with U-[^13^C_6_]-glucose. This may explain why inhibiting mitochondrial translation reduced the frequencies of γδT17 cells and, consequently, psoriatic inflammation in the IMQ model^23^. Furthermore, targeting OXPHOS by inhibiting isocitrate dehydrogenase and pyruvate kinase significantly diminished IL-17A production by skin γδ T cells^25^. Given that FAS supports mitochondrial activity in γδT17 cells, its inhibition can dampen mitochondrial metabolism and limit γδT17 cell function.

OXPHOS generates reactive oxygen species (ROS) as byproducts^66^. Aberrant mitochondrial ROS production impairs mitochondria-mediated OXPHOS^66,67^. Thus, maintaining redox balance is essential for regulating oxidative stress, preserving mitochondrial integrity and optimizing T cell effector functions^68–71^. Our RNA-seq data revealed that γδT17 cells upregulated SLC4A11, a mitochondrial uncoupler located at the inner mitochondrial membrane, under psoriatic conditions^72–74^. SLC4A11 orchestrates ammonia-sensitive H^+^ uncoupling to suppress the production of mitochondrial ROS^72–74^. Ammonia is a byproduct of glutaminolysis^75^. This reaction could generate α-ketoglutarate to enter the TCA cycle and increase OXPHOS, which could, in turn, accelerate the formation of ROS, causing oxidative stress^75^. Ammonia reduces MMP hyperpolarization by activating SLC4A11-mediated H^+^ uncoupling^72–74^. Therefore, the increased expression of SLC4A11 in γδT17 cells under psoriatic conditions could have a key role in lowering the MMP and maintaining mitochondrial function.

Our proteomic data identified that γδT17 cells significantly increased the expression of ME1 and ACLY, the enzymes linking glucose metabolism to de novo FAS^6,30,48–50^, under psoriatic conditions. The glucose isotope tracing experiments ascertained that psoriatic inflammation caused γδT17 cells to increase their rate of FAS to meet their lipid demands for IL-17A production. Accordingly, ACC inhibition with SorA significantly reduced the ability of both murine and human γδT17 cells to produce IL-17A in response to psoriatic conditions in vitro. Therefore, inhibiting ACC1 and other core enzymes of FAS, including ACLY and FASN, represents an attractive therapeutic strategy for psoriasis. Given the potential beneficial effect of pharmacological FAS inhibition in individuals with NAFLD, the development of several natural FAS inhibitors has reached the clinical stage^33^. These studies indicate that hepatocytes can bypass FAS inhibition by scavenging alternative carbon sources in a cell-type-specific manner. For instance, ACLY inhibition increased the expression of ACSS2 in the liver as a compensatory mechanism^33^. Furthermore, a variety of unanticipated secondary effects of hepatic ACC inhibition has also been noted, such as the loss of malonyl-CoA synthesis, leading to increased fatty acid oxidation and gluconeogenesis^76,77^. These clinical implications suggest that any potential compensatory effects should be addressed further to determine whether combination therapies or changes in diet may be required for effective psoriasis management.

We showed that inhibiting ACC1 in γδT17 cells reduced IL-17A production without significantly affecting the viability or physiological functions of the γδ T cells. These findings suggest that ACC1 is a promising target for reducing γδT17-cell-mediated psoriatic inflammation. An important consequence of inhibiting liver ACC is developing hypertriglyceridemia. This condition increases SREBP-1c expression, leading to increased triglyceride release into plasma^78^. By contrast, inhibition of diacylglycerol acyltransferase 2 (DGAT2) downregulated SREBP-1c^79^. Therefore, the co-administration of DGAT2 and ACC inhibitors has been proposed as a potential strategy for the clinical management of raised plasma triglyceride levels for patients with NAFLD^80^. We examined the potential compensatory pathways used by γδT17 cells under psoriatic conditions by investigating the effect of SorA. We found that γδT17 cells downregulated LD-associated proteins upon FAS inhibition. Although the RNA-seq data suggested that γδT17 cells upregulated genes associated with cholesterol metabolism, we observed reduced expression of HMDH protein, controlling the rate-limiting step in the cholesterol synthesis after 24 h of FAS inhibition. The proteomic analysis further indicated that γδT17 cells actively used a compensatory mechanism to release free fatty acids and consumed their stored lipids upon FAS inhibition. Meanwhile, flow cytometric analysis confirmed that γδT17 cells depleted intracellular lipid stores while increasing extracellular lipid uptake to compensate for ACC inhibition. The fact that γδT17 cells can increase lipid uptake following FAS inhibition suggests that they become more dependent on lipid sources (that is, from adipose tissue and dietary lipids)^81^. Therefore, additional diet management should be considered with ACC inhibition as a potential strategy for psoriasis treatment.

In the psoriatic mouse model, the contribution of IL-17-producing dermal γδ T cells to skin inflammation has been acknowledged^10–18,22–26^. Nevertheless, whether or not IL-17-producing γδ T cells have a key role in triggering human psoriasis is still debated^59^. Owing to the low frequency of γδ T cells in human skin, the majority of human γδ T cell studies have been carried out by blood-derived γδ T cells (mainly Vγ9Vδ2^+^)^59^, including our current study. Although IL-17A transcripts were not detected in human γδ T cells, IL-17-committed γδ T cell subsets (by CCR6, RORC and IL23R expression) within Vγ9Vδ2^+^ T cells have been identified in human neonatal cord blood or adult peripheral blood using single-cell RNA-seq analysis^59^. After polarization and stimulation, Vγ9Vδ2^+^ T cells from the peripheral blood of healthy donors can produce detectable levels of IL-17A^58^. We used ex vivo expanded human Vγ9Vδ2^+^ T cells to test the role of FAS in IL-17A production. Whether blood-derived human γδ T cells share a similar metabolic profile with tissue-resident γδ T cells is unclear. Therefore, the importance of the metabolic pathway in skin-resident γδ T cells in psoriatic lesions from patients remains for further investigation.

We showed that γδT17 cells used several approaches, including FAS, lipid uptake and LD storage, to ensure that their lipid demand was met under psoriatic conditions in vitro. Collectively, these results highlight the importance of lipids for γδT17 cells in psoriasis. However, why γδT17 cells rely so heavily on lipids is currently unclear. Our first obvious explanation was that palmitate from FAS was transported into the mitochondria through the carnitine palmitoyltransferase system for fatty acid oxidation^76^. However, using SCENITH analysis and isotope-tracing experiments, we found that γδT17 cells used limited levels of fatty acid oxidation under homeostatic and psoriatic conditions. Mechanistically, FAS and lipid uptake regulate the intracellular lipid composition of γδT17 cells. The availability of different lipid ligands can affect the DNA binding and activity of RORγt in T_H_17 cells^7^. Given that RORγt is also a master transcription factor for γδT17 cells, FAS or lipid uptake could regulate the binding of RORγt to the *Il17a* gene locus in these cells, thus potentially reducing their ability to produce IL-17. In addition, ACC1 inhibition could trigger changes in metabolite availability, potentially affecting post-translational modifications such as acetylation, malonylation and palmitoylation^82^. For instance, ACC1 inhibition could increase acetyl-CoA levels and affect epigenetic regulation through histone acetylation^83–86^.

In addition, FAS provides the building blocks for the synthesis of phospholipids, sphingolipids and glycerolphospholipids required for signalling and membrane structure^33,54^, which could be the underlying machinery of reduced mitochondrial mass, LD formation and trans-Golgi network upon FAS inhibition in γδT17 cells. For instance, palmitate restriction can limit the capacity of membrane biosynthesis to support intracellular cytokine trafficking. The proteomic analysis of SorA-treated γδT17 cells showed a reduced trans-Golgi network, which implicates decreased intracellular cytokine trafficking from the endoplasmic reticulum (ER)–Golgi-intracellular vesicle to deliver IL-17A upon FAS inhibition. Blocking ER–Golgi transport has been shown to interfere with ER functions and thereby lead to ER protein misfolding, accumulation of proteins in the ER, ER stress and ultimately induce unfolded protein response (UPR)^87^. In response to ER stress, cells repress protein biosynthesis to reduce the load of proteins in the ER and, consequently^87^, might inhibit IL-17A translation. Furthermore, proteomic analysis showed that SorA-treated γδT17 cells significantly upregulated DNJB9, a selective repressor of inositol-requiring enzyme 1 (IRE1), one of the main sensors of the UPR^88^, suggesting that an ongoing reaction counteracts with UPR. Therefore, evaluation of the trans-Golgi network and ER stress will reveal whether palmitate supply is required for maintaining the trans-Golgi network following IL-17A translation in response to IL-1β and IL-23. Interestingly, IL-23 activates the IRE1 pathway and enhances T_H_17 responses^89^. Investigating whether DNJB9 agonists can affect IL-17A production in γδT17 by inhibiting IRE1 could provide more insights into the role of ER stress in the regulation of IL-17A.

**Extended Data Fig. 10:**
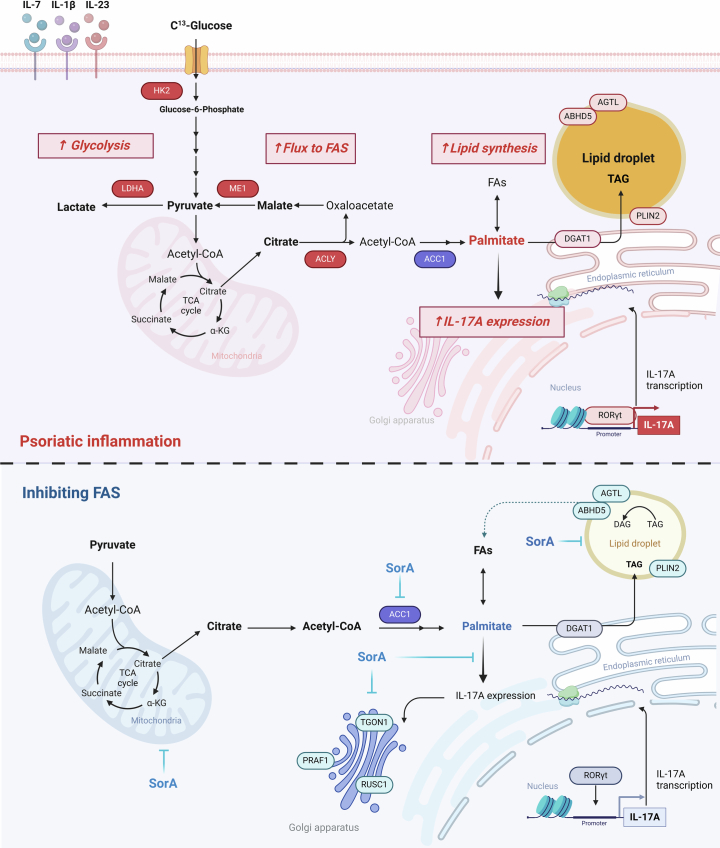
The overview of metabolic reprogramming of γδT17 cells under psoriatic inflammation and SorA-mediated FAS inhibition. γδT17 cells upregulated aerobic glycolysis while reducing mitochondria dependence to support *de novo* FAS to meet their high lipid demand under psoriatic conditions (IL-7 + IL-1β/IL-23) compared to homeostatic conditions (IL-7). Pyruvate produced by the glycolytic pathway is converted into acetyl-CoA in the mitochondria before entering the tricarboxylic acid (TCA) cycle as citrate. Without entering the TCA cycle, citrate can be transported to the cytosol. Upon psoriatic inflammation, γδT17 cells increased the expression of ATP-citrate lyase (ACLY) to link glucose metabolism to FAS by converting citrate to acetyl-CoA and a byproduct, oxaloacetate in the cytosol. Acetyl-CoA can then be used for *de novo* FAS mediated by the rate-limiting enzyme acetyl-CoA carboxylase (ACC1) to synthesize palmitate. Cytoplasmic oxaloacetate is converted to malate and then to pyruvate by malic enzyme 1 (ME1) to rejoin the glycolytic pathway. Meanwhile, ME1, an NADP-dependent enzyme, generates the NADPH required for FAS. FAS-derived palmitate is required for maintaining mitochondrial mass, neutral lipid synthesis, lipid droplet formation (mainly consisting of triacylglycerol, TAG), and IL-17A expression in γδT17 cells in response to psoriatic conditions. FAS inhibition using ACC inhibitor Soraphen A (SorA) reduces mitochondrial mass, intracellular lipid stores, lipid droplet formation, trans-Golgi network, and IL-17A expression while increasing compensatory lipolysis. The illustration was created in BioRender. Kao, Y. (2025) https://BioRender.com/e31v719.

Targeting IL-17A-producing αβ T cells mainly limited psoriatic skin inflammation in the early stages of the disease (that is, days 3–4 after IMQ treatment)^9^, and further targeting γδT17 cells (by genetic deletion) reduced inflammation on day 5 post IMQ treatment. Accordingly, developing a broad-spectrum ACC1 inhibitor to limit the IL-17A-producing capacity of conventional T cells and γδT17 cells represents a promising therapeutic strategy for psoriasis. In summary, our study demonstrated that abolishing ACC1 activity in γδT17 cells effectively reduced IL-17A production upon psoriatic inflammation both in vitro and in vivo (Extended Data Fig. 10). We found that under psoriatic conditions, γδT17 cells preferentially increased the rate of aerobic glycolysis rather than relying on OXPHOS. We performed a proteomic analysis to show that psoriatic conditions selectively supported the pro-inflammatory effector functions of γδT17 cells. Moreover, the γδT17 cells upregulated ACLY, thus linking glucose metabolism to de novo FAS. Stable isotope tracing confirmed that γδT17 cells boosted the rate of glycolysis to support ACC1-mediated FAS under psoriatic conditions. FAS-derived palmitate is required for IL-17A expression in γδT17 cells. Accordingly, FAS inhibition reduced IL-17A production by in vitro-cultured murine and human γδT17 cells. Furthermore, genetic ablation of ACC1 in γδT17 cells reduced the extent of skin inflammation observed in the model of psoriasis. Collectively, our findings suggest that targeting ACC1-mediated FAS in γδT17 cells represents a promising approach for treating psoriasis. Moreover, the pharmacological inhibition of FAS in specific immune cell subsets could open up new avenues for the treatment of other autoimmune and inflammatory conditions.

## Methods

### Mice

Animal experiments were performed with either C57BL/6JRj (Janvier Labs) WT mice or the *Rorc*^ACC1KO^ mouse line, which was generated by crossing *Rorc*^Cre/+^ mice^57^ to *ACC1*^lox/lox^ mice^35^ and maintained on a C57BL/6J genetic background. Their littermate *Rorc*^Cre/wt^*ACCl*^fl/fl^ mice were used as WT controls. Mice were bred and housed in the animal facility of the University Medical Center of the Johannes Gutenberg University of Mainz under specific-pathogen-free conditions. All animal experiments were performed in compliance with the relevant guidelines and regulations for animal welfare by the federal state of Rhineland-Palatinate, Germany. Experiments were done with approval from the Landesuntersuchungsamt Rheinland-Pfalz (individual animal experimentation application no. G19-1-060), and all efforts were made to minimize the potential suffering of the mice. Untreated female and male C57BL/6J and female IL-17A-GFP-reporter (C57BL/6-*Il17a*^*tm1Bcgen*^/J; IL17A-IRES-GFP-KI) mice were used as organ donors for primary γδT17 cell culture.

### Mouse and human γδT17 cell culture

CD27^−^γδ T cells were expanded in vitro by an optimized protocol modified from a previous publication^39^. In brief, the cells were collected from LNs and pre-enriched with biotin-conjugated anti-CD11b antibodies by Streptavidin/Anti-Biotin MicroBeads. Cells were then cultured at 1 × 10^6^ ml^–1^ in IMDM media containing 10% FCS, antibiotics, 10 mM HEPES, 1 mM sodium pyruvate and 50 μM 2-mercaptoethanol in the presence of 5 ng ml^–1^ murine IL-1β/IL-23 and 10 μg ml^–1^ anti-IFNγ (clone XMG1.2; BioXCell) in 96-well round-bottom plates coated with 1 mg ml^–1^ anti-TCR-γδ (clone GL3; Biolegend) for 3 days. Cells were re-seeded on fresh 96-well round-bottom plates at 1 × 10^6^ cells per ml for another 3 days as mentioned above but without anti-TCR-γδ stimulation. This protocol was adapted to have pure CD27^−^γδ T cells as γδT17 cells by sorting out expanded CD27^−^γδTCR^+^ γδT17 cells on day 6 (ref. ^90^). CD27^+^γδ T cells were separated and maintained by 20 ng ml^–1^ IL-7 as a γδIFN control. Sorted γδT17 cells were expanded for 3 days by 20 ng ml^–1^ IL-7. On day 9, the cells were collected and plated at 1 × 10^6^ cells per ml in 96-well U-bottom plates and randomly assigned to be stimulated with cytokines (20 ng ml^–1^ IL-7, 5 ng ml^–1^ murine IL-1β/IL-23) in the presence or absence of indicated SorA concentration. Any cell culture with low viability owing to stress induced by enrichment or sorting or failed experimental procedure was excluded. For human IL-17A-producing Vδ2^+^γδ T cell culture^58^, peripheral blood mononuclear cells were stimulated by 5 µM zoledronate, 50 ng ml^–1^ recombinant human (rh)IL-6, 10 ng ml^–1^ rhIL-23, 10 ng ml^–1^ rhIL-1β and 10 ng ml^–1^ rhTGF-β in IMDM medium (with 10% FCS). On day 6, half of the old medium was removed and replaced with a fresh IMDM medium containing 20 U ml^–1^ rhIL-2 in the presence or absence of the indicated SorA concentration for another 6 days^58^. For intracellular cytokine staining, cells were stimulated with phorbol 12-myristate 13-acetate (0.1 μg ml^−1^; Sigma-Aldrich) and ionomycin (1 μg ml^−1^; Sigma-Aldrich) for 2 h, followed by brefeldin A (5 μg ml^−1^) for 2 h. Data collection and analysis were not performed blind to the conditions of the experiments.

### RNA-seq and bioinformatical analysis

γδT17 cells isolated from mice were enriched as described in the γδT17 cell culture and sorted on day 6 and further expanded for 3 days with IL-7. Following 3 h of incubation with the indicated cytokines, RNA was extracted from γδT17 cells and purified with the RNeasy Plus Micro Kit (Qiagen, cat. no. 74034) according to the manufacturer’s instructions. RNA was quantified with a Qubit 2.0 fluorometer (Invitrogen), and the quality was assessed on a Bioanalyzer 2100 (Agilent) using an RNA 6000 Nano chip (Agilent). Barcoded mRNA sequence libraries were prepared from 100 ng of total RNA (RNA integrity number of >9) using a NEBnext poly(A) mRNA Magnetic Isolation Module and NEBnext RNA Ultra II lib prep kit for Illumina (New England Biosciences, cat. no. E7775) according to the manufacturer’s instructions with a final PCR cycle number of 13. Quality controls were carried out by using Invitrogen’s Qubit HS assay and fragment size was determined on Agilent’s 2100 Bioanalyzer High Sensitivity DNA Assay. Premade libraries were sent to Novogene. After quality control was performed, libraries were pooled and sequenced on Illumina’s NovaSeq 6000 according to effective library concentration and data amount (around 30 million paired-end 150 bp reads per sample). Sequence reads were trimmed for adaptor sequences and further processed using Qiagen’s software CLC Genomics Workbench (v.22.0.1) with default settings for RNA-seq analysis. For statistical analysis, CLC’s count-based ‘Empirical analysis of differential gene expression’ implementing the ‘Exact test’ for two-group comparisons^91^ was applied, and it was filtered for differentially expressed genes by a fold change of >6, difference of >4 and *P* value of <0.05. Reads were aligned to the GRCm38 genome. The heatmaps in Fig. 1a and Supplementary Fig. 1 were prepared using Morpheus (https://software.broadinstitute.org/morpheus). For this purpose, the expression values were shown in the logarithm to the base 2, and hierarchical clustering was performed with one minus Pearson’s correlation, average linkage. The genes with higher than sixfold change are shown in Supplementary Fig. 1 and genes with higher than 12-fold change are in Fig. 1. The pathway enrichment analysis was performed by Enrichr (https://maayanlab.cloud/Enrichr/) using MSigDB’s hallmark gene set and Jensen Compartments^92–94^. The heatmaps of mitochondrial-metabolism-related and glycolytic–lipogenic-associated genes were prepared using SRplot^95^.

### Stable isotope tracing experiment

At the end of the γδT17 cell culture, the cell medium was switched to a glucose-free IMDM medium supplemented with 25 mM U-[^13^C_6_]-glucose in the last 48 h or a lipid-free medium with 100 µM U-[^13^C_16_]-palmitate in the last 24 h. The cells were collected and washed with normal saline. The cell pellets were collected in mass-spectrometry-grade methanol. For metabolite extraction, ice-cold double-distilled H_2_O (containing 1 µg ml^−1^ glutaric acid-d6 as internal standard) and chloroform (containing 30 µg ml^−1^ palmitic acid-d31) were used as previously published^96^. In brief, collected extracts were shaken at 4 °C for 20 min at 1,400 rpm, followed by centrifugation at 4 °C for 5 min at 17,000*g*. For analysis of polar metabolites, the upper phase was transferred to GC vials with micro inserts and evaporated to dryness under vacuum at 4 °C. Dried extracts were derivatized using methoxyamine hydrochloride (20 mg ml^−1^ in pyridine) and MTBSTFA. For palmitate analysis, the lower phase was evaporated to dryness, followed by fatty acid transesterification using 2% H_2_SO_4_ in methanol for 2 h at 50 °C. Fatty acid methyl esters were extracted by sequential addition of saturated NaCl solution and hexane. The upper hexane phase was transferred to glass vials and evaporated to dryness. Dried extracts were dissolved in hexane.

Metabolite separation was performed using a GC 7890 A gas chromatograph (Agilent) in splitless mode equipped with a 30 m DB-35 MS +5 m Duruguard capillary column and acquired on a 5975 MS (Agilent) in selective ion monitoring mode. Data was analysed using Metabolite Detector Software.

### IMQ-induced psoriasis mouse model

Mice (7 weeks old) were shaved and depilated with hair removal cream (Veet) on the back skin 2 days before treatment and then randomly assigned to be treated daily with 50 mg Aldara (containing 5% IMQ; purchased from Meda) or sham cream (without IMQ) on the back skin and 5 mg Aldara or sham cream per ear for both ears for six consecutive days, as previously published^9^. Male mice with scars on the skin from fighting between littermates were excluded from performing the model experiment. For the back skin, skin thickness and disease severity were assessed daily with a scoring system for scaling and erythema in line with the human Psoriasis Area and Severity Index (PASI). Erythema and scaling were scored from zero to four, with zero indicating no severity and four indicating high disease severity. Thickness was scored based on the increase in the back skin thickness on day 0, as previously published^9^.

### Skin digestion protocol

Ears were separated into ventral and dorsal halves and cut into small pieces, followed by enzymatic digestion for 90 min at 37 °C with 4 mg ml^−1^ (1.2 U ml^−1^) collagenase D (Sigma-Aldrich) and 50 U ml^−1^ DNase I (Applichem) in a gentleMACS Dissociator (Miltenyi Biotech).

### Flow cytometry

Single-cell suspension was incubated with an in-house Fc-receptor blocking reagent before staining of surface antigens. Dead cells were excluded with the Live/Dead Fixable Dead Cell Stain Kit (Life Technologies). For analysis of surface markers, cells were stained in PBS containing 0.25% BSA (Roche) and 0.02% NaN_3_ (Carl Roth). For the labelling of murine surface antigens, the following fluorescence-conjugated monoclonal antibodies were used: CD3e (145-2C11; eBiosciences) and γδTCR (GL3; eBiosciences). For intracellular staining of cytokine, cells were stained with IL-17A (eBio17B7; eBiosciences) and Ki-67 (11F6; BioLegend) using the Foxp3/Transcription Factor Fixation/Permeabilization Kit (eBiosciences) according to the manufacturer’s instructions. For mitochondrial mass, membrane potential and neutral lipid measurement, cells were stained with the MitoTracker Green FM Dye, MitoTracker Red CM-H_2_Xros and HCS LipidTOX Red (Thermo Fisher) following the manufacturer’s instructions. As indicated in the respective experiments, cells were stimulated in vitro in the presence of phorbol-12-myristate-13-acetate (0.1 μg ml^−1^; Sigma-Aldrich) and Ionomycin (1 μg ml^−1^; Sigma-Aldrich) for 2 h, followed by incubation for 2 h with brefeldin A (5 μg ml^−1^; eBiosciences) before staining. For lipid uptake measurement, the cells were incubated with BODIPY FL C_16_ (Thermo Fisher) according to the manufacturer’s instructions. Cells were acquired on Cytoflex S (Beckman Coulter) or Cytek Northern Lights (Cytek Biosciences), and data were analysed with FlowJo software (v.10.8.1, Tree Star).

### SCENITH

The SCENITH kit containing all reagents and protocols^45^ was obtained from GammaOmics (http://www.gammaomics.com). After activation of γδ T cells collected from the IMQ model, cells were treated for 15 min at 37 °C, 5% CO_2_ with control (DMSO), 2-DG (100 mM), oligomycin (1 μM) or a combination of both drugs. Puromycin (10 μg ml^–1^) was added for 30 min at 37 °C. Cells were washed in cold PBS and stained with the Live/Dead Fixable Dead Cell Stain Kit (Thermo Fisher Scientific) to exclude the dead cells. After washing with PBS, cells were fixed and permeabilized using the Foxp3/Transcription Factor Fixation/Permeabilization Kit (eBiosciences) according to the manufacturer’s instructions. Intracellular staining of puromycin using the anti-puro monoclonal antibody (1:600, Clone R4743L-E8) was performed by incubating cells for 45 min at room temperature (20 °C)^45^.

### Filter Aided Sample Preparation

For mass spectrometric analysis, cells were collected and washed with PBS. Cell pellets were lysed using a urea-based lysis buffer (7 M urea, 2 M thiourea, 5 mM dithiothreitol (DTT), 2% (w/v) CHAPS). Lysis was further promoted by sonication at 4 °C for 15 min using a bioruptor (Diagenode). After lysis, the protein concentration was determined using the Pierce 660 nm protein assay (Thermo Fisher Scientific) according to the manufacturer´s protocol. Then, 20 µg of total protein was subjected to tryptic digestion using a modified Filter Aided Sample Preparation as previously detailed^97,98^. In brief, samples (corresponding to approximately 20 µg total protein amount) were transferred into spin filter columns (Nanosep centrifugal devices with Omega membrane, 30 kDa MWCO). Afterward, detergents were removed by washing the samples (membrane) three times with a buffer containing 8 M urea and 0.1 M TRIS Base. After reduction and alkylation by DTT and iodoacetamide, excess iodoacetamide was quenched with DTT, and the membrane was washed three times with 50 mM NH_4_HCO_3_. Afterwards, proteins were digested overnight at 37 °C with trypsin (Trypsin Gold, Promega) using an enzyme-to-protein ratio of 1:50 (w/w). After digestion, peptides were recovered by centrifugation and washed with 50 mM NH_4_HCO_3_. Combined flow-through was acidified with trifluoroacetic acid to a final concentration of 1% (v/v) trifluoroacetic acid and then lyophilized. Purified peptides were reconstituted in 0.1% (v/v) formic acid for liquid chromatography–mass spectrometry (LC–MS) analysis.

### LC–MS analysis

LC–MS analyses were performed using an Ultimate 3000 RSLCnano LC system (Thermo Fisher Scientific) coupled to an Orbitrap Exploris 480 instrument platform (Thermo Fisher Scientific). Tryptic peptides were first loaded onto a PEPMAP100 C18 5-µm 0.3 × 5-mm trap column (Thermo Fisher Scientific) and subsequently separated on an HSS-T3 C18 1.8-μm, 75 μm × 250-mm analytical reversed-phase column (Waters Corporation). Mobile phase A contained water with 0.1 % (v/v) formic acid and 3 % (v/v) DMSO. Peptides were separated, running a gradient of 2–35% mobile phase B (0.1 % (v/v) formic acid, 3 % (v/v) DMSO in acetonitrile) over 40 min at a flow rate of 300 nl min^−1^. The total analysis time was 60 min, including the wash and column re-equilibration steps. The column temperature was set to 55 °C. The following settings were used for MS analysis of eluting peptides on the Orbitrap Exploris 480 instrument platform: spray voltage was set to 1.8 kV, the funnel RF level to 40 and the heated capillary temperature was at 275 °C. Data were acquired in data-independent acquisition mode. Full MS resolution was set to 120,000 at *m/z* 200, and full MS automated gain control target to 300% with a maximum injection time of 20 ms. The mass range was set to *m/z* 345–1,250. Fragment ion spectra were acquired with an automated gain control target value of 1,000%. In total, 21 windows with varying sizes (adjusted to precursor density) were used with an overlap of 0.5 Th. The resolution was set to 30,000, and injection time was determined automatically (‘auto mode’). The normalized collision energy was fixed at 27%. All data were acquired in profile mode using positive polarity.

### Data analysis and label-free quantification

MS raw data were processed using DIA-NN (v.1.8)^99^, which applied the default parameters for library-free database search. Data were searched using a custom-compiled database containing UniProtKB and SwissProt entries of the murine reference proteome and a list of common contaminants. For peptide identification and in-silico library generation, trypsin was set as protease, allowing one missed cleavage. Carbamidomethylation was set as a fixed modification, and the maximum number of variable modifications was set to zero. The peptide length ranged between 7 and 30 amino acids. The precursor *m/z* range was set to 300–1,800, and the product ion *m*/*z* range to 200–1,800. As a quantification strategy, we applied the ‘Robust LC (high precision)’ mode with ‘RT-dependent median-based cross-run normalization’ enabled. We used the built-in algorithm of DIA-NN to automatically optimize MS2 and MS1 mass accuracies and scan window size. Peptide precursor false discovery rates (FDRs) were controlled below 1%. In the final proteome datasets, proteins had to be identified by at least two peptides. Statistical analysis of the data was conducted using the Student’s *t*-test, which was corrected by the Benjamini–Hochberg method for multiple hypothesis testing (FDR of 0.01). For functional enrichment of upregulated and downregulated proteins, the KEGG and Gene Ontology enrichment analysis and the FDR were estimated using the freely available software STRING (v.11.5) under a Creative Commons BY 4.0 license (https://string-db.org). Enrichment analysis results were plotted in RStudio.

### Immunofluorescence

Cells were cultured in the presence of brefeldin A (5 μg ml^−1^; eBiosciences) in the last 2 h before staining. After washing with PBS, cells were fixed and permeabilized using the Foxp3/Transcription Factor Fixation/Permeabilization Kit (eBiosciences) according to the manufacturer’s instructions. After the incubation with washing and blocking buffer (10% heat-inactivated goat serum in PBS) for 30 min at room temperature, cells were incubated with monoclonal APC anti-mouse IL-17A antibody (TC11-18H10.13; BioLegend) for 30 min at room temperature. Finally, cells were labelled with NucBlue Live ReadyProbes Reagent (Hoechst 33342; Invitrogen), and LDs were stained with HCS LipidTOX Red (Thermo Fisher) following the manufacturer’s instructions. After incubation for 30 min at room temperature, cells were washed with PBS. Cells were transferred to glass coverslips coated with poly-ʟ-lysine (Sigma-Aldrich) and mounted with ProLong Glass Antifade Mountant (Thermo Fisher). For immunofluorescence, images were collected using a fully motorized Nikon Ti-E with Perfect Focus System (interferometric-based focus maintenance (PFS) equipped with an Agilent high-power MLC400 (150 mW/647 nm; 70 mW/561 nm; 70 mW/488 nm; 25 mW/405 mW). Images were captured with a ×100 objective (numerical aperture, 1.49) using the appropriate lasers and analysed with NIS-Elements software (v.5.2).

### Statistical analysis

Aside from the RNA-seq and proteomic analysis, statistical tests of all the other experiments were performed with GraphPad Prism (v.10) (GraphPad Software). The *P* values were calculated using a Student’s *t*-test, one-way ANOVA or two-way ANOVA, as indicated. The *P* values were considered significant at *P* < 0.05. Data distribution was assumed to be normal but this was not formally tested.

### Ethics approval

All animal experiments were performed in compliance with the relevant guidelines and regulations for animal welfare by the federal state of Rhineland-Palatinate, Germany. The experimental protocols were approved by the Landesuntersuchungsamt Rheinland-Pfalz (individual animal experimentation application no. G19-1-060), and efforts were made to minimize animal suffering.

### Materials availability

Unique resources generated in this study are available upon reasonable request, although they may require the completion of a Material Transfer Agreement.

### Reporting summary

Further information on research design is available in the Nature Portfolio Reporting Summary linked to this article.

## Supplementary information

Supplementary Information Supplementary Figs. 1–4

Reporting Summary

## Data Availability

The datasets generated and analysed during this study are available from the following public repositories. The RNA-seq data generated in this study were deposited to the Gene Expression Omnibus (GEO) under accession number GSE256512. The proteomic data generated in this study were deposited to the public repository ProteomeXchange and iPOST. The accession numbers for the raw data are PXD050505 for ProteomeXchange and JPST002976 for jPOST. Any additional information required to reanalyse the data reported in this paper is available from the lead contact upon request
