# Supplementary material for: Metabolic reprogramming of interleukin-17-producing γδ T cells promotes ACC1-mediated de novo lipogenesis under psoriatic conditions
Source: Nat Metab. 2025 May 13;7(5):966–84. doi: 10.1038/s42255-025-01276-z (PMC12116387; doi:10.1038/s42255-025-01276-z)
Supplement: Supplementary file 1 — Supplementary Figs. 1–4 [file 42255_2025_1276_MOESM1_ESM.pdf]

nature metabolism

Supplementary information

https://doi.org/10.1038/s42255-025-01276-z

Metabolic reprogramming of interleukin-17-producing γδ T cells promotes ACC1-mediated de novo lipogenesis under psoriatic conditions

In the format provided by the authors and unedited

Supplementary figures

Matabolites	Derivatve	Quantification Ions	SIM window (min)	Fragment SumFormula	IL-7 #1	IL-7+ IL-1β/IL-23 #1	IL-7 #2	IL-7+ IL-1β/IL-23 #2	IL-7 #3	IL-7+ IL-1β/IL-
Alanine_260	2TBDMS	260 261 262 263 264	8.80 - 9.20	C11H26O2NSi2	0.01745	0.020532222	0.01896	0.029210502	0.0155	0.027832507
Aspartate_418	3TBDMS	418 419 420 421 422 423	15.30-15.85	C18H40NO4Si3	0.06831	0.066988438	0.04273	0.073393409	0.0721	0.061432246
Citrate_591	4TBDMS	591 592 593 594 595 596 597	18.70-20.00	C26H55O7Si4	0.01775	0.020829205	0.02635	0.042498064	0.012	0.039272436
Fumarate_287	2TBDMS	287 288 289 290 291 292 293	11.85-12.30	C12H23O4Si2	0.00187	0.044797683	0.00332	0.00611838	0.0568	0.055716567
Glutamate_432	3TBDMS	432 433 434 435 436 437 438 439 440	16.40-16-80	C19H42O4NSi3	0.25092	0.354060248	0.13976	0.301884746	0.2278	0.282295182
Glycine_246	2TBDMS	246 247 248 249 250	9.20 - 9.55	C10H24O2NSi2	0.05372	0.05759285	0.06139	0.074974213	0.0571	0.053309111
Isoleucine_302	2TBDMS	302 303 304 305 306 307	10.90-11.20	C14H32NO2Si2	0.01436	0.015930293	0.03216	0.034657892	0.0076	0.04726931
Lactate_261	2TBDMS	261 262 263 264 265	8.00-8.80	C11H25O3Si2	0.21036	0.346022733	0.33573	0.947924525	0.0969	0.440971438
Leucine_302	2TBDMS	302 303 304 305 306 307	10.50-10.90	C14H32NO2Si2	0.01686	0.018257785	0.01907	0.025883468	0.019	0.033826062
Malate_419	3TBDMS	419 420 421 422 423 424	14.95-15.30	C18H39O5Si3	0.00718	0.008454233	0.00541	0.013904947	0.0035	0.011065487
Serine_390	3TBDMS	390 391 392 393 394 395 396	13.30-14.00	C17H40NO3Si3	0.00611	0.007426206	0.00643	0.009968944	0.0039	0.010582759
Succinate_289	2TBDMS	289 290 291 292 293 294	11.85-12.30	C12H25O4Si2	0.01164	0.011730952	0.01709	0.02081603	0.0104	0.01942119
Valine_288	2TBDMS	288 289 290 291 292	10.00-10.50	C13H30NO2Si2	0.00773	0.008451223	0.01107	0.015948441	0.0059	0.017705263

Supplementary Fig. 1| Metabolite abundances of *in vitro*-expanded γδT17 cells. Cells were sorted on day 6 and cultured with interleukin (IL)-7 for 3 days. On day 9, cells were re-seeded and stimulated with IL-7 either alone (homeostatic conditions) or combined with IL-1β and IL-23 (psoriatic conditions) for 48 h. Cells were collected on day 11, metabolites were extracted and measured by GC-MS in selected ion monitoring mode (SIM). Normalized metabolite abundance data of biological replicates from three independent experiments are shown in the table.

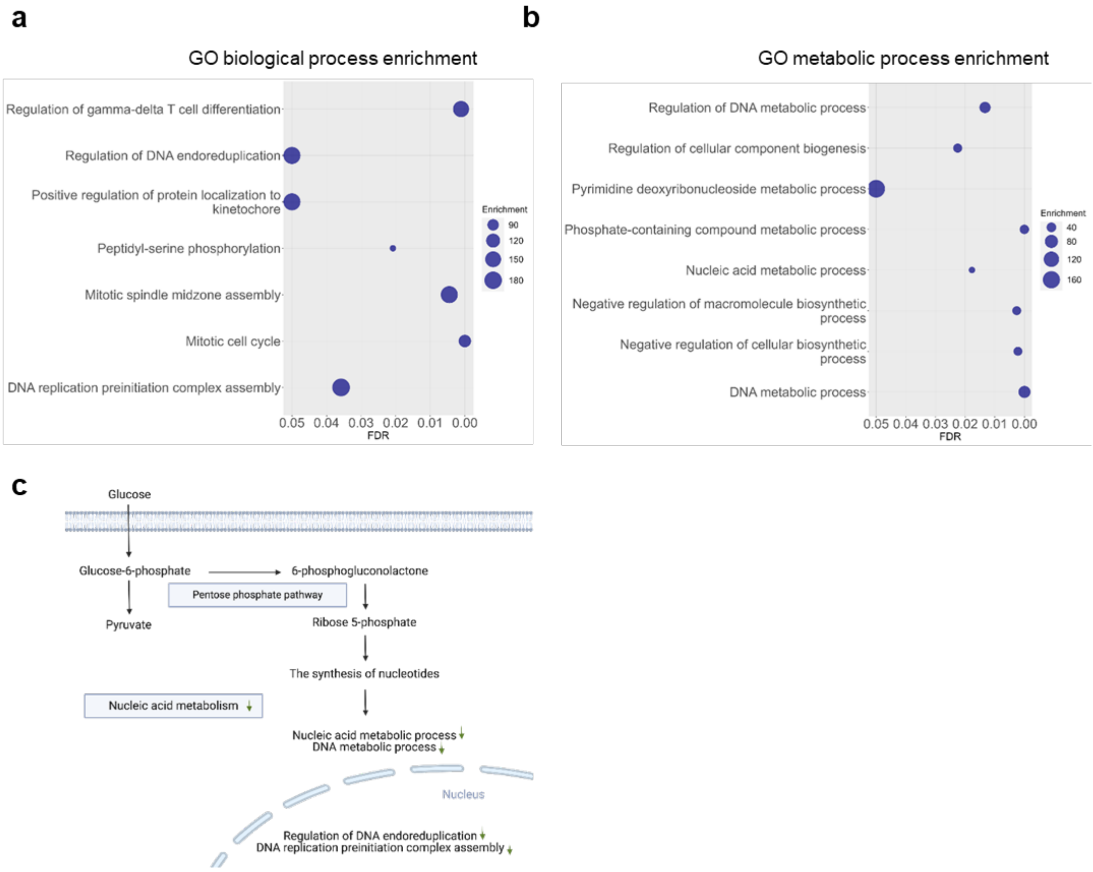

Supplementary Fig. 2| γδT17 cells downregulate the DNA metabolic process under psoriatic conditions. On day 9, *in-vitro*-expanded γδT17 cells were re-seeded and stimulated with IL-7 alone (homeostatic conditions) or combined with IL-1β and IL-23 (psoriatic conditions) for 24 h before proteomic analysis. GO pathway enrichment analysis of biological process **a** and metabolic process **b**; proteins downregulated (blue) under psoriatic versus homeostatic conditions are shown. **c**, Schematic model of the pathway analysis with downregulated proteins under psoriatic versus homeostatic conditions. The enrichment scores and false discovery rate (FDR) were estimated using STRING. Data from four independent experiments were collected for the proteomic analysis, and *p*-values were obtained using the two-sided Benjamini-Hochberg corrected t-test. $p<0.01$ indicates statistically significant differences. **c**, Schematic model of pathway analysis of differentially expressed proteins under psoriatic versus homeostatic conditions was created in BioRender. Kao, Y. (2025) https://BioRender.com/s59i821.

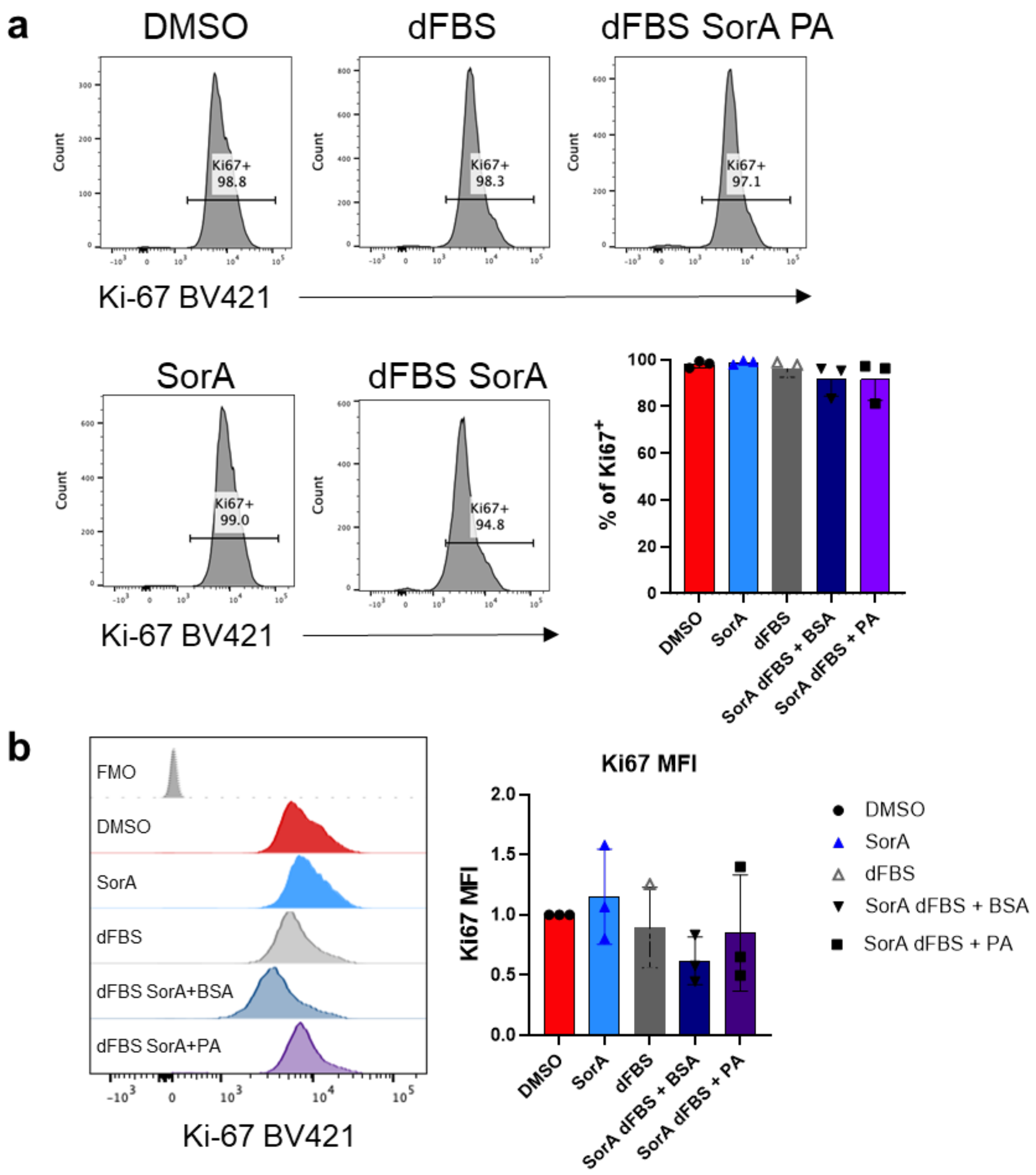

Supplementary Fig. 3| FAS inhibition does not affect γδT17 proliferation in the *in vitro* culture of γδT17 cells for 24 h. *In vitro*-expanded γδT17 cells were sorted on day 6 and cultured with IL-7 for 3 days. On day 9, cells were re-seeded and stimulated with either IL-7 (homeostatic conditions) or a combination of IL-1β and IL-23 (psoriatic conditions) for 24 h. **a,** The percentage, and **b,** the mean fluorescence intensities (MFIs) of Ki-67 expression in γδT17 cells after 24-h culture under DMSO control, SorA, or delipidated medium (dFBS) conditions as indicated. Pooled means from three independent experiments are shown. Error bars represent standard deviation (SD), and *p*-values were determined using the one-way ANOVA. ns $p>0.05$.

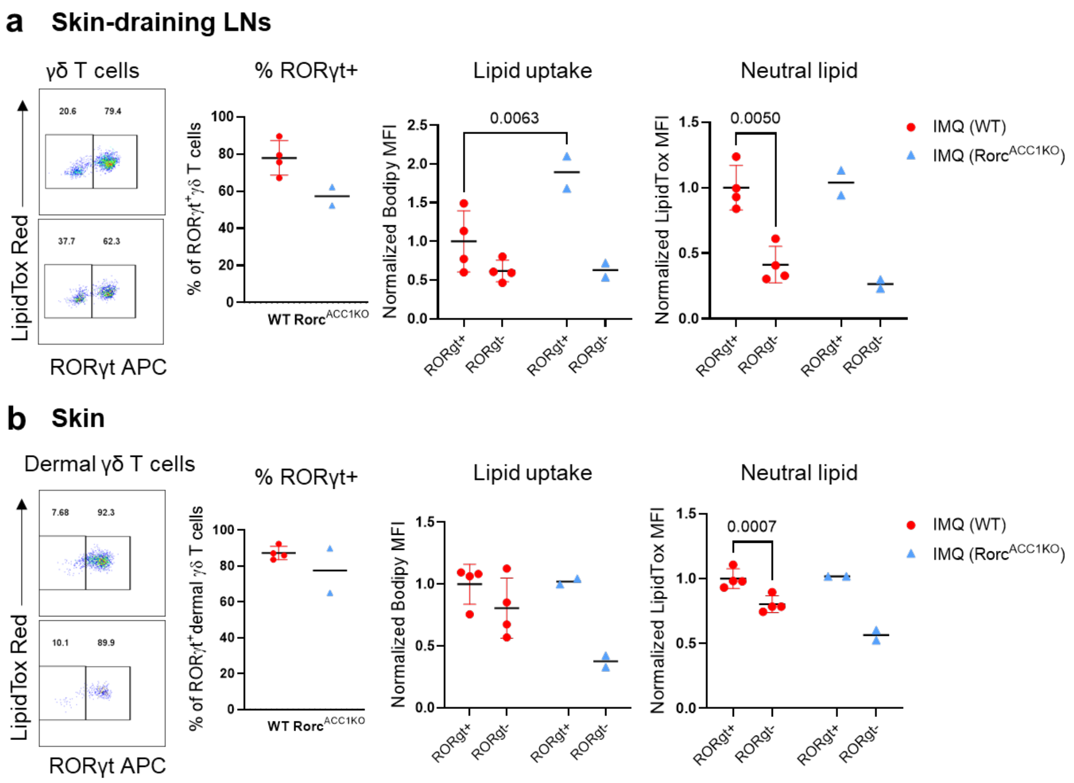

Supplementary Fig. 4| Genetic ablation of ACC1 in RORγt⁺ γδT17 cells increases compensatory lipid uptake in the skin-draining LNs. The ears and back skin of wild-type (WT) or $Rorc^{ACC1KO}$ model mice were treated topically with imiquimod (IMQ) for six consecutive days. **a**, Flow cytometry gating strategy and for identifying $ROR\gamma t^{+}$ γδ T cells to evaluate lipid uptake and intracellular neutral lipid content within skin-draining LNs of the treated ear areas on day 6. **b,** Flow cytometry gating strategy and for identifying $ROR\gamma t^{+}$ among dermal γδ T cells to evaluate lipid uptake and intracellular neutral lipid content within skin areas of the treated ear areas on day 6. Error bars represent standard deviation (SD), and *p*-values were obtained using the two-way ANOVA. ns $p>0.05$.
