# Supplementary material for: Metabolic reprogramming of interleukin-17-producing γδ T cells promotes ACC1-mediated de novo lipogenesis under psoriatic conditions
Source: Nat Metab. 2025 May 13;7(5):966–84. doi: 10.1038/s42255-025-01276-z (PMC12116387; doi:10.1038/s42255-025-01276-z)
Supplement: Supplementary file 2 — Reporting Summary [file 42255_2025_1276_MOESM2_ESM.pdf]

nature portfolio

Corresponding author(s): Tim Sparwasser and Yu-San Kao

Last updated by author(s): Apr 10, 2025

Reporting Summary

Nature Portfolio wishes to improve the reproducibility of the work that we publish. This form provides structure for consistency and transparency in reporting. For further information on Nature Portfolio policies, see our Editorial Policies and the Editorial Policy Checklist.

Statistics

For all statistical analyses, confirm that the following items are present in the figure legend, table legend, main text, or Methods section.

n/a	Confirmed	
☐	☒	The exact sample size (*n*) for each experimental group/condition, given as a discrete number and unit of measurement
☐	☒	A statement on whether measurements were taken from distinct samples or whether the same sample was measured repeatedly
☐	☒	The statistical test(s) used AND whether they are one- or two-sided<br>*Only common tests should be described solely by name; describe more complex techniques in the Methods section.*
☐	☒	A description of all covariates tested
☐	☒	A description of any assumptions or corrections, such as tests of normality and adjustment for multiple comparisons
☐	☒	A full description of the statistical parameters including central tendency (e.g. means) or other basic estimates (e.g. regression coefficient) AND variation (e.g. standard deviation) or associated estimates of uncertainty (e.g. confidence intervals)
☐	☒	For null hypothesis testing, the test statistic (e.g. *F*, *t*, *r*) with confidence intervals, effect sizes, degrees of freedom and *P* value noted<br>*Give P values as exact values whenever suitable.*
☒	☐	For Bayesian analysis, information on the choice of priors and Markov chain Monte Carlo settings
☐	☒	For hierarchical and complex designs, identification of the appropriate level for tests and full reporting of outcomes
☐	☒	Estimates of effect sizes (e.g. Cohen's *d*, Pearson's *r*), indicating how they were calculated

Our web collection on statistics for biologists contains articles on many of the points above.

Software and code

Policy information about availability of computer code

Data collection	Metabolite separation was performed using a GC 7890A gas chromatograph (Agilent) in splitless mode equipped with a 30 m DB-35 ms+ 5 mDuruguard capillary column and acquired on a 5975 mass spectrometry (Agilent) in selective ion monitoring mode. Flow cytometry data were acquired on Cytoflex S (Beckman Coulter) or Cytek Northern Lights (Cytek Biosciences,F remont,C A). Liquid chromatography-mass spectrometry (LC-MS) analysis for proteomic data was collected using an Ultimate 3000 RSLCnano LC system (Thermo Fisher Scientific) coupled to an Orbitrap Exploris 480 instrument platform (Thermo Fisher Scientific).
Data analysis	Metabolite data was analyzed using Metabolite Detector Software (https://md.tu-bs.de/). RNA-seq data were analyzed with CLC Genomics Workbench and heatmaps were prepared by Morpheus (https://software.broadinstitute.org/ morpheus) and SRplot (https:// www.bioinformatics.com.cn/en). RNA-seq data enrichment analysis was performed by Enrichr (https:// maayanlab.cloud/Enrichr/). Proteomic raw data were processed using DIA-NN (version 1.8),a nd enrichment analysis results were analyzed with STRING (version 11.5) and plotted via R studio (R studio, version 4.4.1, Posit, P BC). Flow cytometry data were analyzed using FlowJo software (version 10.8.1, Tree Star). Images obtained by confocal microscopy were analyzed by NIS-Elements software (version 5.2). Graph Pad Prism (Version 10) was used for statistical analysis. All analyses are described in the methods.

For manuscripts utilizing custom algorithms or software that are central to the research but not yet described in published literature, software must be made available to editors and reviewers. We strongly encourage code deposition in a community repository (e.g. GitHub). See the Nature Portfolio guidelines for submitting code & software for further information.

Data

Policy information about availability of data

All manuscripts must include a data availability statement. This statement should provide the following information, where applicable:

- Accession codes, unique identifiers, or web links for publicly available datasets
- A description of any restrictions on data availability
- For clinical datasets or third party data, please ensure that the statement adheres to our policy

The datasets generated and analyzed during this study are available from the following public repositories. The RNA-seq data generated in this study were deposited to the Gene Expression Omnibus (GEO) under accession number GSE256512. The proteomic data generated in this study were deposited to the public repository ProteomeXchange and iPOST. The accession numbers for the raw data are PXD050505 for ProteomeXchange and JPST002976 for jPOST.

Research involving human participants, their data, or biological material

Policy information about studies with human participants or human data. See also policy information about sex, gender (identity/presentation), and sexual orientation and race, ethnicity and racism.

Reporting on sex and gender	N/A
Reporting on race, ethnicity, or other socially relevant groupings	N/A
Population characteristics	N/A
Recruitment	N/A
Ethics oversight	N/A

Note that full information on the approval of the study protocol must also be provided in the manuscript.

Field-specific reporting

Please select the one below that is the best fit for your research. If you are not sure, read the appropriate sections before making your selection.

☒ Life sciences ☐ Behavioural & social sciences ☐ Ecological, evolutionary & environmental sciences

For a reference copy of the document with all sections, see nature.com/documents/nr-reporting-summary-flat.pdf

Life sciences study design

All studies must disclose on these points even when the disclosure is negative.

Sample size	No statistical analysis was used to pre-determine the sample size. Pilot experiments were used to estimate the required sample sizes and referred to previously published results with similar experimental methods [6, 9, 21].
Data exclusions	Observed outliers and failed experiments were excluded. If a data point is more than 2 standard deviations above or below the mean is considered an outlier. Any cell culture with low viability due to stress induced by enrichment or sorting or failed experiment procedure was excluded.
Replication	All experiments were repeated as described in the figure legends.
Randomization	Samples were randomly allocated into experimental groups.
Blinding	The majority of the experiments were not blinded as preparation of cell culture and readout measurement were carried out by the same investigator. The cell culture and metabolite measurement were performed by different investigators.<br>The results in Figure 6 were performed blinded by revealing the genotypes after completing the score determination and analysis.

Reporting for specific materials, systems and methods

We require information from authors about some types of materials, experimental systems and methods used in many studies. Here, indicate whether each material, system or method listed is relevant to your study. If you are not sure if a list item applies to your research, read the appropriate section before selecting a response.

Materials & experimental systems

n/a	Involved in the study
☐	☒ Antibodies
☒	☐ Eukaryotic cell lines
☒	☐ Palaeontology and archaeology
☐	☒ Animals and other organisms
☒	☐ Clinical data
☒	☐ Dual use research of concern
☐	☐ Plants

Methods

n/a	Involved in the study
☒	☐ ChIP-seq
☐	☒ Flow cytometry
☒	☐ MRI-based neuroimaging

Antibodies

Antibodies used

Purified anti-mouse TCR g/d Antibody (1:500, clone: GL3, Biolegend, cat#118101); CD11b Monoclonal Antibody (1:100, clone Ml/70, Biotin, Bioscience, cat#B-0112-82); lnVivoMAb anti-mouse IFNγ (10 µg/ml, clone: XMG1.2, Bio X Cell, cat#BE0055); CD3e Monoclonal Antibody (1:200, 145-2C11, PerCP-Cyanine5.5/APC, eBiosciences, cat#45-0031-82/17-0031-82); PE anti-mouse TCR Vγ4 Antibody (1:100, clone UC3-10A6, Biolegend, cat#137706); TCR gamma/delta Monocional Antibody (1:100, clone GL-3, eBioGL3 (GL-3, GL3), eFluor 450, eBioscience, cat#48-5711-82); IL-17A Monoclonal Antibody (1:200, clone eBio17B7, PE- Cyanine7, eBioscience, cat#25-7177-82); CD27 Monoclonal Antibody (1:200, clone LG.7F9, PE, eBioscience, cat#11-0271-82); Ki-67 (1:100, clone 11F6, BV421, Biolegend cat#151208); APC anti-mouse IL-17A Antibody (1:200, clone TC11-18H10.13; Biolegend, cat# 506915).

Validation

All antibodies are commercially available and validated by the manufacturers.

Biolegend antibodies: https://www.biolegend.com/en-us/quality/quality-control
Biolegend employs a comprehensive approach to antibody validation, analyzing 1-3 target cell types with single- and multi-colour analysis to encompass positive and negative cell types. Upon confirming specificity, each new lot is required to match the intensity of the in-date reference lot, with the brightness (MFI) evaluated across both positive and negative populations to ensure consistency. Furthermore, quality control testing, including a series of titration dilutions, is conducted for every lot.

eBioscience reagents for immunology, multicolor flow cytometry, and biological system analysis are now part of Thermo Fisher Scientific.
Thermo Fisher Scientific antibodies: https://www.thermofisher.com/de/de/home/life-science/antibodies/invitrogen-antibodyvalidation.html
Thermo Fisher Scientific tests each antibody using different methods, including flow cytometry, Immunoprecipitation-Mass Spectrometry Antibody Validation, Knockout and Knockdown Antibody Validation, Independent Antibody Validation, Peptide Array Antibody Validation, Cell Treatment, Neutralization Antibody Validation, Relative Expression Antibody Validation, and SNAP-ChIP Antibody Validation. The precise validation method for each antibody is outlined in its respective antibody datasheet.

The XMG1.2 antibody from InVivoMab is a neutralizing antibody and validated by western blot: https://bioxcell.com/pub/media/tds/BE0055-TDS.pdf

Animals and other research organisms

Policy information about studies involving animals; ARRIVE guidelines recommended for reporting animal research, and Sex and Gender in Research

Laboratory animals

Animal experiments were performed with either 7 to 10 week-old C57BL/6JRj (Janvier Labs) WT mice or the RorcACC1KO mouse line. The RorcACC1KO mouse line was generated by crossing RorcCre/+ mice [57] to ACCllox/lox mice [35] and maintained on a C57BL/6J genetic background. Their littermate RorcCre/wtACClfl/fl mice were used as WT controls. Mice were bred and housed in the animal facility of the University Medical Center of the Johannes Gutenberg-University of Mainz under specified pathogen-free conditions. All mice were kept on a 12-h light-dark-cycle (24°C, air humidity 55%).

Wild animals

No wild animals were used in the study.

Reporting on sex

Untreated female and male C57BL/6J and female IL-17A-GFP-reporter (C57BL/6-1117atm1Bcgen/J; IL17A-IRES-GFP-KI) mice were used as organ donors for primary γδT17 cell culture. For, IMQ-induced psoriasis mouse model, mice were shaved and depilated with hair removal cream (Veet®, Reckitt Benckiser Group, Slough, England) on the back skin 2 days before the treatment and then daily treated with 50 mg Aldara (containing 5% IMQ, purchased from Meda [Solna, Sweden]) or sham cream (without IMQ) on the back skin and 5 mg Aldara or sham cream per ear for both ears for six consecutive days, as previously published [9]. Male mice with scars on the skin from fighting between littermates were excluded from performing the model experiment.

Field-collected samples

No field collected samples were used in the study.

Ethics oversight

All animal experiments were performed in compliance with the relevant guidelines and regulations for animal welfare by the federal state of Rhineland-Palatinate, Germany. Experiments were done with approval from the Landesuntersuchungsamt Rheinland-Pfalz (individual animal experimentation application no. G19-1-060), and all efforts were made to minimize the potential suffering of the mice.

Note that full information on the approval of the study protocol must also be provided in the manuscript.

Plants

Seed stocks	N/A
Novel plant genotypes	N/A
Authentication	N/A

Flow Cytometry

Plots

Confirm that:

☒ The axis labels state the marker and fluorochrome used (e.g. CD4-FITC).

☒ The axis scales are clearly visible. Include numbers along axes only for bottom left plot of group (a 'group' is an analysis of identical markers).

☒ All plots are contour plots with outliers or pseudocolor plots.

☒ A numerical value for number of cells or percentage (with statistics) is provided.

Methodology

Sample preparation	Single-cell suspension was incubated with in-house Fe-receptor blocking reagent before staining of surface antigens. Dead cells were excluded by the LIVE/DEAD Fixable Dead Cell Stain Kit (Life Technologies). For analysis of surface markers, cells were stained in PBS containing 0.25% BSA (Roche, Mannheim, Germany) and 0.02% NaN3 (Carl Roth GmbH+Co.KG, Karlsruhe, Germany). For the labeling of murine surface antigens, the following fluorescence-conjugated monoclonal antibodies were used: CD3e (145-2C11; Biosciences, San Diego, CA) gdTCR (GL-3; Biosciences, San Diego, CA). For intracellular staining of cytokines or antigens, cells were stained with IL-17A (eBio17B7; eBiosciences, San Diego, CA) Ki-67 (11F6, Biolegend, San Diego, CA); APC anti-mouse IL-17A Antibody (TC11-18H10.13; Biolegend, San Diego, CA) using the Foxp3/Transcription Factor Fixation/Permeabilization Kit (Biosciences, San Diego, CA) according to the manufacturer's instructions. For mitochondrial mass, membrane potential, and neutral lipid measurement, cells were stained with the MitoTracker'"' Green FM Dye, MitoTracker'" Red CM-H2Xros, and HCS LipidTox Red (Thermo Fisher, Waltham, MA) following the manufacturer's instructions. As indicated in the respective experiments, cells were stimulated in vitro in the presence of phorbol-12-myristate-13-acetate (PMA) (0.1 µg/mL; Sigma-Aldrich, St. Louis, MO) and lonomycin (1 µg/mL; Sigma-Aldrich, St. Louis, MO) for 2 h followed by incubation for 2 h with Brefeldin A (5 µg/ml; eBiosciences, San Diego, CA) before staining. For lipid uptake measurement, the cells were incubated with BODIPY FL C16 (Thermo Fisher) according to the manufacturer's instructions.
Instrument	For flow cytometry, Cells were acquired on Cytoflex S (Beckman Coulter) or Cytek Northern Lights (Cytek Biosciences, Fremont, CA), and data were analyzed with FlowJo software (Tree Star).
Software	Flow cytometry data were analyzed using FlowJo software (version 10.8.1, Tree Star). Graph Pad Prism (Version 10) was used for statistical analysis. All analyses are described in the methods.
Cell population abundance	Post-sorting samples contained at least 90% of the desired cell population for further analysis.
Gating strategy	We excluded the debris by FSC/SSC and doublets by SSC-H/SSC-A and FSC-H/FSC-A. We used live/dead dye to exclude the dead cells.<br>We define cell populations: IL-17A production in Vγ4+γδ T cells (CD45+γδTCR+Vγ4+), Vγ4-γδ T cells (CD45+γδTCR+Vγ4-), CD4+ T cells (CD45+αβTCR+CD4+γδTCR-), CD8+ T cells (CD45+αβTCR+CD8+γδTCR-), double-negative αβ T cells (CD45+αβTCR+CD4-CD8-γδTCR-), dendritic epidermal T cells (DETCs, CD45+γδTCRhigh), non-T cells within treated-skin.<br>We provided gating strategies for dyes and antigens in the figures.

☒ Tick this box to confirm that a figure exemplifying the gating strategy is provided in the Supplementary Information.
